# Early Secure Attachment as a Protective Factor Against Later Cognitive Decline and Dementia

**DOI:** 10.3389/fnagi.2019.00161

**Published:** 2019-07-04

**Authors:** Emilie Walsh, Yvonne Blake, Alessia Donati, Ron Stoop, Armin von Gunten

**Affiliations:** ^1^Service of Old Age Psychiatry, Department of Psychiatry, Lausanne University Hospital (CHUV), Lausanne, Switzerland; ^2^Center for Psychiatric Neurosciences, Department of Psychiatry, Lausanne University Hospital (CHUV), Lausanne, Switzerland

**Keywords:** attachment, protective factor, aging, cognitive decline, dementia

## Abstract

The etiology of neurodegenerative disorders such as dementia is complex and incompletely understood. Interest in a developmental perspective to these pathologies is gaining momentum. An early supportive social environment seems to have important implications for social, affective and cognitive abilities across the lifespan. Attachment theory may help to explain the link between these early experiences and later outcomes. This theory considers early interactions between an infant and its caregiver to be crucial to shaping social behavior and emotion regulation strategies throughout adult life. Furthermore, research has demonstrated that such early attachment experiences can, potentially through epigenetic mechanisms, have profound neurobiological and cognitive consequences. Here we discuss how early attachment might influence the development of affective, cognitive, and neurobiological resources that could protect against cognitive decline and dementia. We argue that social relations, both early and late in life, are vital to ensuring cognitive and neurobiological health. The concepts of brain and cognitive reserve are crucial to understanding how environmental factors may impact cognitive decline. We examine the role that attachment might play in fostering brain and cognitive reserve in old age. Finally, we put forward the concept of affective reserve, to more directly frame the socio-affective consequences of early attachment as protectors against cognitive decline. We thereby aim to highlight that, in the study of aging, cognitive decline and dementia, it is crucial to consider the role of affective and social factors such as attachment.

## Introduction

As the prevalence of dementia continues to increase, the fear of cognitive decline is becoming a central preoccupation in the elderly population. Multiple genetic and environmental factors play a role in the development of dementia, and a great deal of scientific interest is currently focused on identifying relevant risk and protective factors. Various types of dementia exist, including Alzheimer's Disease, Vascular Dementia, Lewy Body Dementia, and Frontotemporal Dementia. Additionally, Mild Cognitive Impairment is used to describe an intermediate state between healthy aging and dementia. Mild Cognitive Impairment is characterized by cognitive deficits and related dysfunction not severe enough to be diagnosed as dementia, though the presentation can vary considerably among individuals (Winblad et al., 2004). Even in healthy aging, it is normal to observe some decline in several but not all cognitive domains. Whereas working memory, episodic memory, processing speed, and some aspects of short-term memory are typically impacted, other abilities such as general language or basic conceptual functions tend to be spared.

In the search for protective factors against cognitive decline and dementia, the potential role of early attachment has been largely overlooked. Many studies have demonstrated that a supportive social environment in old age can protect against the progress of cognitive decline (Bennett et al., 2006; Gow et al., 2007). However, early social support may also have long lasting psychosocial, cognitive and neurobiological consequences. Furthermore, a safe early social environment could offer protection against cognitive decline through its effects on the establishment of particular emotions regulation strategies.

Early attachment refers to the quality of the interaction between the child and the primary caregiver. By repetition, the child progressively integrates these interactions into mental representations, which allow for the establishment of long-term attachment patterns. These patterns will then determine social behavior and emotional regulation strategies throughout adult life, and will be particularly useful in times of stress as they may help the individual to regain a feeling of comfort and well-being (Bretherton and Munholland, 1999; Cassidy, 2000; Waters and Waters, 2006). Prompted by multiple experiences of loss, separation, and vulnerability associated with aging, these established attachment patterns may pervade a person's perceptions, feelings and attitudes, both in healthy aging and in the presence of cognitive disorders (Bradley and Cafferty, 2001; Perren et al., 2007). Therefore, we believe that it is highly important to study attachment patterns in this particular population.

We will begin this review by introducing the concept of attachment and it's impact on an individual's social relations. We will then discuss how quality social relations may protect against cognitive decline. Next, we will examine how attachment might affect cognitive and neurobiological development of various brain structures and systems and the consequences this may have for later cognitive decline, as a vast literature has shown that some early environmental factors can significantly modulate and drive the interplay of numerous processes involved in brain maturation (Gluckman et al., 2008; Brummelte, 2017). Finally, we will discuss how early attachment might contribute to brain and cognitive reserve, which play a role in protecting against cognitive decline. We will consider evidence from different developmental fields of study including epigenetics, neurobiology and psychology.

## Attachment, Social Factors, Cognitive Decline, and Dementia

“*Man is by nature a social animal”* Aristotle, *Ethique à Nicomaque*.

For many species, living in a group ensures a sustained food supply, protection against predators, reproduction and the opportunity to learn survival skills. Mammalian infants in particular are highly dependent on social interactions for their day-to-day needs. In humans, deprivation and neglect can have devastating and long-lasting consequences on children's intellectual and emotional development. One example of this is hospitalism syndrome, which is a form of developmental stunting first described by Spitz (1945). This syndrome refers to the severe physical and psychological retardation observed in children separated from their mothers for several months during their first year of life. Spitz (1947) observed that this deprivation of affection caused a progressive deterioration of the personality that eventually led, in more than one third of chronically hospitalized children, to marasmus and death by the end of the second year. This evidence demonstrates that social contact is fundamentally necessary to life itself and led Bowlby, who is considered the father of attachment theory, to assume that human beings have an innate need of interpersonal relations and social support (Bowlby, 1988).

Weak social ties and the experience of early maltreatment may affect both physical (Uchino, 2006; Reblin and Uchino, 2008) and mental health (Antonucci, 2001) across the lifespan. Studies in the elderly population have shown associations between early maltreatment or absent or weak social relations, both early and current, and higher incidence of cardiovascular diseases (Orth-Gomér et al., 1993; Rosengren et al., 2004; Burg et al., 2005), worse prognosis in breast cancer (Kroenke et al., 2006, 2013, 2017), increased risk of mortality (Seeman et al., 1993; Seeman, 2000; Santini et al., 2015; Kauppi et al., 2017), late-life depression and suicide risk (Dennis et al., 2005; Cacioppo et al., 2006; Fiori et al., 2006; Glass et al., 2006; Jardim et al., 2018, 2019; Novelo et al., 2018). Moreover, some studies suggest that, for elderly individuals, poverty or lack of social relations or integration may favor cognitive decline and increase the risk of developing dementias such as Alzheimer's disease (Tilvis et al., 2004; Bennett et al., 2006; Gow et al., 2007; James et al., 2011).

As early interactions with the caregiver may be the basis through which a person integrates and forges their social abilities, attachment theory is a valuable candidate to explain to which degree a person feels driven to seek proximity with others in various contexts. Furthermore, attachment theory could also predict the degree to which an individual is able to benefit from this social proximity, which could subsequently affect the preservation (or deterioration) of their cognitive functioning.

### Attachment and Social Functioning

Based on the results of ethological studies, Bowlby (1969) argues that a child's attachment behaviors toward their caregiver are of vital necessity. Ainsworth (1979), Bowlby (1982) and subsequent authors have conceptualized attachment as a behavioral system. However, more recent evidence suggests that attachment is unlikely to rely on the functioning of any single identifiable neurological system, but probably results from the interplay of various social and motivational systems in the brain (Panksepp, 1998). Thus, throughout this review we will use the term “attachment system” to refer to behaviors, representations and psychological processes related to attachment. To avoid any confusion, we also define the term “behavior” as the way in which one acts or conducts themselves, especially toward others.

Bowlby assumes that attachment behaviors are regulated by an innate motivational system whose main function is to establish a physical proximity with the attachment figure in case of real or perceived danger, or anxiety-provoking situations in general. Factors that trigger this need for safety can be either environmental (such as an unfamiliar stimulus or the rapid and threatening approach of an object) or directly related to the child's internal state (such as tiredness, hunger or illness) (Bowlby, 1969). Children adopt certain behaviors and signals to alert their caregivers to their needs. These signals, that include, among others, crying or calling the caregiver, essentially reflect the search for proximity with the attachment figure during the occurrence of stressful situations. These attempts to achieve proximity are called “primary strategies.” The manner in which those close to the child respond to the child's behavior will guide the development of attachment styles; which will in turn form the basis for establishing effective internal models that will govern the feelings, expectations, and behavior of the individual in their relationships. Once the child's needs have been satisfied and eased, it resumes its activities (Ainsworth et al., 1978).

Since birth, the child begins to develop a repertoire of attachment behaviors that aim to catch or keep the attention of their caregiver. Depending on the effectiveness of these primary strategies the child will be more or less inclined to adapt their behavior and develop “secondary strategies.” Secondary strategies include, on the one hand, avoidance of closeness and, on the other hand, energetic or exaggerated attempts to seek proximity or support. However, if the caregiver meets the child's needs in response to the primary strategies, the proximity search may cease as its goal has been achieved and the child can relax. If this positive and reassuring interaction consistently occurs throughout the early years of development, the child should become “securely attached.” However, proximity-seeking behavior is not triggered exclusively by stressful or unpleasant states. For example, Trevarthen et al. (2006) emphasize the child's natural inclination for joyful social engagement, such as playing. Such positive interactions also contribute to the building and strengthening of secure attachment ties.

In the case of predominantly inconsistent or unavailable responses from the caregiver, the child will increasingly tend to adopt secondary strategies (Main, 1990). These strategies are constructed based on the child's assessment of whether reconciliation with the caretaker is possible, and how best to maintain a sustainable relationship with them. The use of secondary strategies reflects the development of “insecure attachment”. Insecure attachment behavior may be triggered not only through the unavailability of the caregiver in times of need, but also in the case of inappropriate responses from them (Trevarthen et al., 2006).

As mentioned, secondary strategies can take two different forms. If the child is faced with an unavailable caregiver, they inhibit their primary strategies and adopt an avoidant attitude. A child who predominantly demonstrates secondary strategies of this type will be referred to as “avoidantly attached.” If, on the contrary, access to the inconsistent caregiver seems possible, the child will respond by exaggerating and distorting attachment behaviors, resulting in crying and clinging. A child who predominantly adopts secondary strategies of this type will be referred to as “anxiously attached”. Due to the difficulty of classifying all children into the three categories of attachment behavior discussed thus far, “disorganized” attachment style has been proposed as a fourth category (Main and Solomon, 1986). This type of insecure attachment is characterized by contradictory responses, oscillating between exaggerated and inhibited attachment behaviors.

The repetition of secure or insecure strategies will gradually be internalized and generate an interpersonal expectation of the attachment figure's availability toward the self and the availability of the self toward others. These internalized expectations, called Internal Working Models (IWMs), will generalize through various relationships and contribute to the establishment of internal regulation mechanisms. IWMs shape the representation one has of oneself and others, guiding behaviors, thoughts and coping strategies to be adopted in social interactions or in particularly stressful times (Main et al., 1985; Bretherton and Munholland, 1999; Cassidy, 2000). Thus, the attachment style defines an individual's emotion regulation abilities, which will in turn modulate their internal state and subsequent behaviors.

Despite their prototypical aspect (Sroufe, 1983; Collins and Read, 1994) and their influence on adult relationships (Carver, 1997; Miljkovitch and Cohin, 2007; Miljkovitch, 2009), IWM are not frozen representations. They can be modified through various life experiences and an individual's panel of behavioral or emotional responses can be enlarged (Vaughn et al., 1979; Bretherton, 1995). Nevertheless, some of the initial structure remains, and the first attachment experiences continue to steer individuals throughout adulthood (Waters et al., 2000; Carlson and Egeland, 2004; Grossmann et al., 2005; Groh et al., 2014).

More recent work by Panksepp (1998) highlights the neuro-affective mechanisms that may underlie the activation of attachment strategies. According to Panksepp, mammals are equipped with seven distinct but integrated neuro-emotional systems, i.e., FEAR, RAGE, PANIC/GRIEF, PLAY, SEEKING, LUST, and CARE. The SEEKING system has no direct object in the sense that it is considered to be a generalized motivational system, which “provides the arousal and energy that activates our interest in the world around us” and, as such, it drives the other six systems (Solms and Turnball, 2002, p. 115). Panksepp's theory assumes that social attachment is built on evolutionarily more ancient systems. For example, ancient pain mechanisms would underlie feelings of separation distress. Thus, various neuro-emotional systems described by Panksepp, such as the PANIC, CARE, and PLAY systems, are likely to be involved in the construction of attachment bonds.

Panksepp considers attachment bonds to be intrinsically related to the neural circuits of distress activated by separation, meaning that PANIC circuits are important for the development of social interactions. The distress triggered by real or felt separation will activate the PANIC system, which will induce the need to seek proximity and social support. In this context, to restore the homeostatic balance, the SEEKING system will promote specific behaviors, including vocalizations, in order to favor social reunion. The behaviors or signals from a distressed child will in turn activate the parent's PANIC circuits, which will then activate the CARE system and lead the parent to provide protection and reassurance to their infant. However, the quality of the parent's CARE system depends on the caring experiences they have themselves gone through and internalized during their own childhood. Therefore, if the parent has experienced significant trauma, a state of anxiety or affective instability (disturbance) may remain, which will in turn influence the way in which the parent perceives their child's needs and responds to them. As previously discussed, the quality of the parent's response to their child will play a crucial role in shaping the child's attachment style (Panksepp, 1998).

The PLAY system is of particular importance as an early pro-social system due to the high levels of positive affect it evokes and its role in the refinement of social interactions (both by promoting the integration of social rules and by building empathy and trust) (Burgdorf et al., 2010; Watt, 2017). The first manifestation of social play arises as early as 2 months of age in humans. Through brief visual and auditory exchanges, the child and the primary caregiver experience their first social interactions by adjusting their attention and expressions based on one another's responses (Schore, 2001). Through these interactive situations and the joyful, pleasant feeling the child experiences, they progressively internalize the possibility of shared attention and practice adjusting their social behaviors and responses to one another (Kestly, 2014). In the section Influence of Early Attachment on Neurobiological and Cognitive Development, we will briefly examine the neurobiological links between separation distress, PLAY and attachment, in which neuropeptides such as endogenous opioids, oxytocin, vasopressin and prolactin are likely to play a critical role (Panksepp et al., 1997; Panksepp, 1998).

### Effects of Social Relations on Cognitive Decline

Social context later in life is important in protecting against cognitive decline. Early attachment may be influential in determining both the availability of social support later in life as well as the degree to which an individual is able to benefit from such support. By maintaining social activities, a person will be engaged in stimulating and complex interactions, which require a variety of cognitive skills. Consequently, social interactions may in turn slow cognitive decline and the development of dementia (Seeman et al., 2001; Wang et al., 2002; Fratiglioni et al., 2004; Beland et al., 2005; Amieva et al., 2010; Qiu et al., 2010; Dickinson et al., 2011; Ellwardt et al., 2013). The influence of such psychosocial factors on cognitive abilities could be due to the internal feeling of comfort conveyed by social support, which may help to lower the level of stress and improve the capacity to face difficult life events (Wilson et al., 2011). Stress, anxiety, and/or depression may therefore induce or favor cognitive decline and the risk of developing later dementia (Beaudreau and O'Hara, 2008; Dotson et al., 2010; Gulpers et al., 2016; Freire et al., 2017).

Seeking proximity and support is a common coping strategy in the case of fear or stress (Zeidner and Endler, 1996; Mikulincer et al., 2003). Attachment style not only influences an individual's evaluation of a threat and moderates their need for social support, but it also shapes the strategies and effort they employ to seek the proximity needed to return to a feeling of well-being (Mikulincer and Florian, 1998; Collins and Feeney, 2000). For instance, as opposed to insecurely attached individuals, securely attached individuals tend to naturally and effectively seek proximity and rely on social support when facing a stressor (Larose and Bernier, 2001; Mikulincer and Florian, 2003), and experience positive effects and reduced stress when recalling the memories of a partner or an available attachment figure (McGowan, 2002; Rowe and Carnelley, 2003). In line with attachment theory, Siedlecki et al. (2014) assume that the feeling of contentment brought by satisfying relationships depends on the concrete sense of having people to turn to in case of need, but also on the expectation that relying on someone else is comforting.

Social relationships can be appraised from an objective or subjective point of view. For example, relational support can be objectively assessed by considering the size of the network, the frequency of contacts and the types of social ties available (marital, family, friends, and caretakers). From this perspective, different studies have revealed that living alone with no or few personal ties (Crooks et al., 2008) as well as being single or widowed (Helmer et al., 1999; Håkansson et al., 2009; Feng et al., 2014; Sundström et al., 2016) increases the risk of cognitive decline and dementia relative to people living with their spouse or partner. Attachment security has also been related to a larger social network in elderly individuals (Fiori et al., 2011).

Though some studies have found that a greater social network significantly reduces the risk of developing dementia (Tilvis et al., 2004; Wilson et al., 2007; Crooks et al., 2008), Amieva et al. (2010) showed that the quality of support impacts the occurrence of later dementia more than its quantity. This suggests that the subjective aspect of social support, i.e., the manner in which a person perceives the quality of the support they receive, is paramount. To distinguish the influence of objective and subjective social support on the onset of later dementia, Amieva et al. (2010) examined a variety of social network characteristics. They investigated six different aspects, namely marital status, number of ties, nature of the social network, satisfaction with network interactions, perception of being understood/misunderstood and reciprocity in the relationship. The results revealed that perceived social support variables had a more significant effect on the risk of developing later dementia than quantitative social support variables. Experiencing satisfaction in relationships reduced the risk of later developing dementia by 23% and by 55% when the participants reported that they received more support than they gave.

Perception of social support is likely to vary according to attachment style. Securely attached individuals demonstrate more optimistic life appraisal (Mikulincer and Florian, 1995; Berant et al., 2001; Shorey et al., 2003), more positive representations of others (Collins and Read, 1990; Simpson, 1990; Baldwin et al., 1996), more positive self-esteem and self-worth (Bartholomew and Horowitz, 1991; Brennan and Morris, 1997; Mikulincer et al., 2004), and more effective coping strategies (Cassidy, 1994; Simpson et al., 1996; Gross and John, 2003). Taken together, these factors may favor the maintenance of cognitive and affective availability, which may sustain an individual's capacity to invest themselves in daily life activities and in their social network. This investment will in turn protect against cognitive decline.

Individuals with insecure attachment profiles will be less able to access fruitful and supportive relationships (Simpson and Rholes, 2017). Insecure attachment has been connected to greater levels of depression, anxiety, psychosomatic illness and feeling of loneliness (Hazan and Shaver, 1990; Carnelley et al., 1994). Avoidant attachment is mainly characterized by self-reliance, as the other is perceived as dismissive and non-supportive. In order to maintain self-reliance, an avoidant individual will suppress painful memories and feelings associated with relationships from consciousness. This will help them maintain a low level of stress by avoiding threatening emotions, but this will also deprive the person from the emotional benefits another person can provide in times of stress. Therefore, when facing a threatening or emotional situation, an avoidant person will inhibit proximity needs and divert his attention toward other interests or goals (Mikulincer et al., 2003; Mikulincer and Shaver, 2007).

Conversely, an anxious attachment style is characterized by self-defeating representations and a pattern of anxiety-driven behaviors accompanied by pessimistic thoughts of others, considered as unable to provide sufficient support (Collins and Read, 1994). Anxious people tend to increase their thoughts and feelings of despair and unworthiness by focusing their attention on negative and painful aspects of themselves, their relationships, or situations (Kobak et al., 1993). Therefore, they display increased and possibly exaggerated attention and support-seeking behaviors (Cassidy and Berlin, 1994). These individuals rarely feel sufficiently reassured and an enduring feeling of dissatisfaction in their social relations remains.

Some studies have focused on the feeling of loneliness to explain the link between perceived social support and the occurrence of dementia. Loneliness is a subjective feeling of social isolation. It describes the distress a person experiences when their social relationships are perceived as unsatisfactory both in terms of quantity and especially quality. Consequently, some people may feel lonely even though they are socially engaged (Ayalon, 2016). Although social isolation and a lack of social engagement have been shown to increase the risk of cognitive decline and dementia (Bassuk et al., 1999; Helmer et al., 1999; Wang et al., 2002), loneliness appears to have even stronger effects on the emergence of these pathologies (Wilson et al., 2007; Holwerda et al., 2014). Thus, people who experience loneliness are twice as likely to develop Alzheimer's disease as those who do not feel lonely (Wilson et al., 2007). These results are consistent with those of Holwerda et al. (2014), who showed that the perceived absence of social relations and support were independently related to increased risk of cognitive decline over a 3-year follow-up. Furthermore, the effect of perceived social isolation on subsequent cognitive decline was significantly stronger than the effect of objective social isolation. Two longitudinal studies also showed that loneliness contributes to increased cognitive decline over periods of 10 and 4 years, respectively (Tilvis et al., 2004; Shankar et al., 2013).

Recent studies on young adults as well as elderly individuals also showed that insecurely attached people were more prone to experiencing loneliness than securely attached people, who express more satisfaction about the support they perceive and receive (Bernardon et al., 2011; Akdogan, 2017; Spence et al., 2018). Although a relationship between loneliness and insecure attachment has been demonstrated, the specific implications for anxious and avoidant attachment styles remain unclear. Loneliness and depression both contributed to worsening elderly people's cognitive abilities over the course of a 12-year longitudinal study (Donovan et al., 2017). The authors suppose that the feeling of loneliness may contribute to a state of emotional distress, which in turn may promote the emergence of a depressive syndrome.

Depression, both early and later in life, has been consistently linked to cognitive decline and later dementia (e.g., Jorm, 2000; Leonard, 2007; Byers and Yaffe, 2011; Da Silva et al., 2013; Zahodne et al., 2013; Donovan et al., 2014; Geda et al., 2014; Santos et al., 2016). Depression also seems to be fundamentally connected to attachment, with a vast body of work demonstrating that attachment-related early life stress can predispose an organism to depression (e.g., Heim and Nemeroff, 1999; Pryce et al., 2005; Heim and Binder, 2012; Nemeroff, 2016; Taillieu et al., 2016; Cecil et al., 2017). Indeed, Watt and Panksepp (2009) conceptualize depression as arising from an evolutionarily preserved “shutdown mechanism” resulting from protracted separation distress in early life. A comprehensive examination of the literature linking early attachment to depression, on the one hand, and depression to cognitive decline, on the other hand, is beyond the scope of this review. However, it is worth considering that depression may play a mediating role in the influence that early attachment could have on later cognitive decline. Furthermore, many of the neurobiological mechanisms which link attachment and separation distress to depression (see Watt and Panksepp, 2009 for a review), will also come forward in our discussion of the neurobiological links between attachment and cognitive performance and decline later in the text.

Thus far, we have examined the importance of social and affective relationships for psychological development and the maintenance of general well-being into old age. We have attempted to clarify this relationship through the lens of attachment theory. However, the impact of these social exchanges, and indeed of attachment processes, can also be observed at the neurobiological and cognitive level.

## Influence of Early Attachment on Neurobiological and Cognitive Development

It is now widely accepted that the early childhood environment plays a crucial role in neurobiological and cognitive development (Brummelte, 2017). For infant mammals, the most meaningful aspect of their environment is their social context as it is through interactions with their caregivers that their needs are met (Kundakovic and Champagne, 2015; Chen and Baram, 2016). Early life social stress can therefore leave an enduring imprint on brain connectivity and, thus, cognition and behavior (Fareri and Tottenham, 2016). In this section we present data from both human and animal literature, as studies in animals are uniquely able to offer insights into the causal mechanisms whereby attachment and early life stress forge neurocognitive development. Indeed, Bowlby (1958) himself strongly recommended an ethological approach to the study of attachment.

The first years of life are characterized by remarkable cerebral plasticity (Diamond, 2013) during which an individual's experiences can greatly influence the development and specialization of synaptic networks (Fox et al., 2010; Kolb et al., 2012). Brain maturation over the course of childhood involves the development of connectivity patterns through synaptic stabilization, pruning and branching of dendrites and myelinisation (Bale et al., 2010; Regev and Baram, 2014). During this period of maturation, early attachment relations may have a significant impact on later cognitive abilities. Indeed, securely attached children appear to demonstrate better cognitive skills than insecure children do (De Ruiter and van IJzendoorn, 1993; Van IJzendoorn, 1995; Moss and St-Laurent, 2001;West et al., 2013).

As previously mentioned, the emotional, relational and cognitive development of the child is linked very early on to the quality of the investment of, and safety of its relationships with, its caregivers. The postnatal period appears as a moment of high sensitivity of brain development to stress. Especially if it is chronic and associated with prolonged secretion of cortisol, stress is likely to leave a neurobiological trace that can affect the entire life of the individual. Changes in brain architecture can lead to impaired intellectual, physical and affective development. Early toxic stress can cause subsequent hyper-reactivity to minor stresses with mental and physical consequences that persist into adulthood. Hence the importance of appropriate caregiver-child relationships that do not provoke excessive stress is clear.

In addition, Bowlby (1982) and Ainsworth et al. (1978) proposed that secure attachment would promote an individual's drive to explore their environment, a behavior which is critical to learning and cognitive development. This link with the exploration system may therefore constitute another mechanism by which early attachment can influence later cognitive abilities.

### Epigenetic Processes: Mediators of Early Life Experiences on Neurobiological Function

Epigenetic processes may constitute mechanisms through which early attachment impacts later cognition as they allow environmental factors to long lastingly alter gene expression, and hence the phenotype, without altering the DNA sequence (Champagne, 2008).

Animal research has demonstrated that epigenetic regulators such as DNA methylation and acetylation of histones are crucial mechanisms by which the mother pup relationship can influence brain processes later in life (Gervai, 2009; Kundakovic and Champagne, 2015). DNA methylation refers to a chemical modification of the DNA bases, where higher levels of methylation usually lead to lower rates of gene transcription and consequently gene functioning (Allis and Jenuwein, 2016; Ein-Dor et al., 2018). Acetylation of histones, on the other hand, leads to greater levels of gene transcription (Zentner and Henikoff, 2013).

Weaver et al. (2004) provide a clear example of an epigenetic mechanism by which maternal care during early development can affect adult behavior. They demonstrated that differences in licking, grooming and nursing behaviors of rat mothers led to differences in the DNA methylation of the glucocorticoid receptor (GR) gene promotor in the hippocampus. In particular, the offspring of low licking and grooming (LG) mothers show increased DNA methylation of the GR gene lasting into adulthood, leading to reduced hippocampal GR expression which in turn leads to an elevated hypothalamic-pituitary-adrenal (HPA) axis response to stress (Weaver et al., 2004). This paper forms part of a body of work by Meaney and his colleagues which clearly demonstrates that variations in rodent maternal care have important consequences for HPA functioning, and subsequently also for various cognitive abilities (e.g., Liu et al., 1997, 2000; Caldji et al., 2000; Meaney, 2001; Champagne et al., 2003, 2008). For example, under conditions of stress, low LG pups demonstrate impaired spatial memory when compared with high LG pups (Liu et al., 2000). However, low LG pups show comparatively enhanced hippocampal long-term potentiation under conditions of stress, which has been linked to enhanced contextual fear conditioning (Champagne et al., 2008).

Subsequently, this same research group demonstrated a relationship between childhood abuse and epigenetic regulation of the human hippocampal glucocorticoid receptor (NR3C1) expression (McGowan et al., 2009). Increased methylation of NR3C1 has also been linked to attachment avoidance in humans (Ein-Dor et al., 2018), while Bosmans et al. (2018) showed a relationship between increased NR3C1 methylation and anxious attachment. NR3C1 methylation may lead to less efficient down-regulation of the HPA axis, thereby constituting a mechanism by which insecure attachment can affect emotion regulation and the stress response across the lifespan (Ein-Dor et al., 2018).

### Brain Structures and Systems That Are Affected by Early Life Experiences

A number of studies have shown that early stress can lead to lasting changes in the activity, connectivity, and volume of various brain structures like the amygdala, hippocampus, and prefrontal cortex (PFC), as well as neuroendocrine, neurotransmitter and neuropeptide systems such as the hypothalamic-pituitary-adrenal (HPA) axis and the oxytocinergic system (see Chen and Baram, 2016, for a recent review). The functioning of these structures and systems is closely related. Thus, changes to any one of them can have direct and indirect consequences for the functioning of other brain structures and systems relevant for the development of cognitive impairment in later life (Chen and Baram, 2016). Here, we focus particularly on those changes that may have consequences for the development of cognitive impairment in later life.

#### The Hippocampus

The hippocampus is a structure with a prolonged post-natal developmental trajectory. It is both highly sensitive to the effects of early-life stress and critical to later cognition as it plays a central role in memory processes (Chen and Baram, 2016). Research in rodents suggests that early life stress impacts hippocampal synaptic plasticity and impairs performance on hippocampus-driven memory tasks such as object recognition and object location into late adulthood (e.g., Brunson et al., 2005; Hulshof et al., 2011; Molet et al., 2016; Pillai et al., 2018; see Derks et al., 2017 for a recent review). Impoverished dendritic trees in the rodent hippocampus following early life stress have also been linked to impaired memory later in life (Ivy et al., 2010; Molet et al., 2016). These changes in dendritic trees likely lead to a reduced number of functional synapses and may progressively worsen with age (Brunson et al., 2005; Ivy et al., 2010). Furthermore, reduced hippocampal volume has been observed both in rodents exposed to early life stress (Molet et al., 2016) as well as in humans who experienced childhood adversity (Buss et al., 2007; Hanson et al., 2015; Teicher and Samson, 2016). Quirin et al. (2010) report reduced hippocampal cell density in insecurely attached individuals. In contrast, maternal support in early childhood has been positively associated with hippocampal volume (Kim et al., 2010; Luby et al., 2016). Rifkin-Graboi et al. (2015) also report a positive relationship between maternal support and hippocampal volume, and between maternal support and hippocampal connectivity to other limbic regions, most importantly the amygdala.

#### The Amygdala

In contrast to the typically observed reduced hippocampal volume following early life stress, severe childhood stress has been linked repeatedly with increased volume of the human amygdala (Mehta et al., 2009; Tottenham et al., 2010; Lupien et al., 2011; Tottenham, 2012; Davidson and McEwen, 2013; Pechtel et al., 2014). Furthermore, Lyons-Ruth et al. (2016) found that an insecure attachment in infancy predicted greater amygdala volume in adulthood and Coplan et al. (2014) found that early life stress was also associated with amygdala enlargement in macaques. The amygdala is a limbic structure that undergoes developmental changes throughout childhood and is critical to the expression and regulation of fear and anxiety. Thus, it is not surprising that early life stress and insecure attachment can impact its development (Tottenham, 2012; Fareri and Tottenham, 2016). In the rodent amygdala, early life stress leads to various changes including dendritic hypertrophy in the basolateral nuclei (Eiland et al., 2012), altered connectivity (Johnson et al., 2018), and increased activity in response to stress later in life (Sanders and Anticevic, 2007; Malter Cohen et al., 2013). In each case, these neurobiological changes are accompanied by enhanced anxiety and impaired fear regulation. Conversely, appropriate early maternal care (as indexed by high as opposed to low licking, grooming and nursing behaviors) has been associated with both differences in amygdala development and reduced fearfulness later in life (Caldji et al., 1998).

Altered amygdala functioning and connectivity has also been observed following early life stress in humans. Such alterations, which often involve increased amygdala reactivity as well as increased amygdala volume, have furthermore been associated with behavioral changes such as enhanced anxiety across the lifespan (Tottenham et al., 2010, 2011; McCrory et al., 2011; Pechtel and Pizzagalli, 2011; Burghy et al., 2012; Gee et al., 2013; Malter Cohen et al., 2013; Fan et al., 2015; McLaughlin et al., 2015; Lyons-Ruth et al., 2016). However, Hanson et al. (2015) report smaller amygdala volumes in children exposed to various types of early life stress. It is likely that amygdala responses to early life stress are non-linear, and differential outcomes later in life may be related to differences in the timing and severity of early life stress (Pechtel et al., 2014; Callaghan and Tottenham, 2015; Hanson et al., 2015).

#### The Prefrontal Cortex

The prefrontal cortex (PFC), which is critical to cognitive and behavioral control, can be significantly affected by early life stress (e.g., Van Harmelen et al., 2010, 2014; McEwen and Morrison, 2013; Yang et al., 2015; Demir-Lira et al., 2016). In humans, the PFC undergoes a particularly protracted maturation process, with certain time-windows during early infancy, childhood and adolescence being important for different aspects of this brain area's development (Diamond, 2002; Gogtay et al., 2004). Adverse life events and attachment experiences during any of one of these time-periods may therefore have a lasting impact on PFC functioning. In fact, numerous reports link attachment to the development of cerebral structures, and particularly areas of the PFC, since early stress interferes with brain maturation and, thus, cognition as well as the development of the attachment system (Kraemer, 1992; Schore, 1996; Gunnar and Quevedo, 2007; Belsky and de Haan, 2011). For example, early life stress has been shown to lead to changes in the dendritic density and morphology of medial PFC neurons and to corresponding functional deficits in rodents (Bock et al., 2005; Monroy et al., 2010; Chocyk et al., 2013; Yang et al., 2015; Soztutar et al., 2016). In both rodents and humans, early life stress has also been linked to altered connectivity of the PFC to limbic brain regions such as the hippocampus and the amygdala (e.g., Burghy et al., 2012; Demir-Lira et al., 2016; Reincke and Hanganu-Opatz, 2017; Johnson et al., 2018). Adults who experienced childhood emotional maltreatment show both reduced volume and reduced activation of the medial PFC (Van Harmelen et al., 2010, 2014). Correspondingly, severe early life stress can result in deficient executive control (Hostinar et al., 2012), to which the medial PFC seems to be key (Ridderinkhof et al., 2004).

Executive functions are cognitive processes that permit action initiation or inhibition and allow for adapted responses to new or problematic situations (Hughes, 2011). Executive functions such as working memory, inhibition and flexibility can be considered as cognitive self-regulation mechanisms (Zelazo et al., 2004; Diamond et al., 2007; Liew, 2012). The early family environment can influence the development of executive functions (Bernier et al., 2010, 2012; Matte-Gagné and Bernier, 2011). Indeed, at first, a child relies on the caregiver for stimulation and regulation, but little by little, they internalize these processes to form their own self-regulation system (Calkins and Leerkes, 2004; McClelland et al., 2010). The quality of these first exchanges, paired with the maturation of cerebral structures and the developing capacity to self-regulate, work together to support the development of executive capabilities. By building the potential to control and inhibit impulses, to learn how to direct attention and to modulate emotions (Zimmerman and Schunk, 2001; Crugnola et al., 2011; Panfile and Laible, 2012), self-regulation allows the child to initiate voluntary and controlled actions (Calkins and Leerkes, 2004; Diamond et al., 2007; McClelland et al., 2010). Parental stimulation, encouragement, sensitivity, and support for autonomy all tend to enhance the development of subsequent working memory, flexibility and attention skills (Bibok et al., 2009; Bernier et al., 2010; Matte-Gagné and Bernier, 2011; Mezzacappa et al., 2011; Hammond et al., 2012; Clark et al., 2013; Hopkins et al., 2013).

#### The HPA Axis

Disruption of the hypothalamo-pituitary-adrenal axis (HPA axis) is likely to drive molecular mechanisms leading to altered hippocampal synaptic plasticity following early life stress (Ivy et al., 2010; Derks et al., 2017). The HPA axis drives a chain of neuroendocrine events in response to stress, starting with the release of corticotropin releasing factor (CRF or CRH) from the hypothalamus. CRF is subsequently the primary trigger for adrenocorticotropic hormone (ACTH) secretion by the anterior pituitary gland, which in turn triggers the systemic release of glucocorticoids by the adrenal gland (Bale and Vale, 2004). Changes in CRF release also appear to be implicated in the process whereby early life stress may impair the structural development of the PFC (Yang et al., 2015). The HPA axis is crucial for controlling the regulation of cortisol, the stress hormone, and therefore the behavioral stress response, throughout life (Rincón-Cortés and Sullivan, 2014). Dysregulation of this axis is a frequently observed consequence of early stress.

Corticosteroid hormones (mainly cortisol in humans and corticosterone in rodents) bind to mineralocorticoid receptors (MR) and glucocorticoid receptors (GR) that are expressed abundantly in limbic structures and are important for the transcriptional regulation of certain genes. Fluctuations in the levels of such hormones are thereby able to cause changes in gene expression (De Kloet et al., 2005). Such gene-environment interactions demonstrate how over-excitation of the HPA axis can lead to increased stress-susceptibility and how specific neurological changes can have important consequences for the development of other brain regions and systems (De Kloet et al., 2005).

#### Growth Hormone and the Insuline-Like-Growth Factor Axis (GH-IGF-1)

Various neurobiological signaling mechanisms that are likely to be influenced by early life stress may also play a role in the extent to which cognition is reduced later in life. For example, abnormally heightened HPA axis activity may lead to the suppression of the growth hormone-insulin-like growth factor axis (GH-IGF-1). Indeed, HPA axis dysregulation due to psychosocial causes in institutionalized children has been linked to suppression of the GH-IGF-1 axis and consequent growth failure (Johnson and Gunnar, 2011). Interestingly, IGF-1 is not only crucial to normal tissue growth, but also affects neuroplasticity and cognitive brain functioning throughout the lifespan (Aleman and Torres-Aleman, 2009; Dyer et al., 2016). A decrease in IGF-1 has been strongly implicated in age-related cognitive decline and has been identified as a potential risk factor for dementia (Sonntag et al., 2013; Ashpole et al., 2015; Doi et al., 2015; Quinlan et al., 2017; Frater et al., 2018). As social deprivation in childhood can lead to deficits in IGF-1 (see Johnson and Gunnar, 2011 for a review), suppression of IGF-1 may constitute a pathway whereby adverse attachment experience related to early life stress can exacerbate age-related cognitive decline. Furthermore, IGF-1 extracellular signaling genes are upregulated by juvenile rough-and-tumble play in rats (Burgdorf et al., 2010). Such rough-and-tumble play is considered a highly positive social interaction, and the underlying PLAY system is conceptually linked to early attachment (see section on Attachment and Social Functioning). Therefore, it seems that social deprivation could suppress IGF-1 signaling on the one hand, while on the other hand, positive social interaction could promote IGF-1 signaling, with potentially important consequences for cognitive functioning during aging.

#### Neuropeptide Signaling

Early attachment is likely to have important consequences for neuropeptide signaling throughout the course of life. For example, oxytocin (OT) seems to play a central role in the neurobiological basis of attachment across species. This hormone behaves like a neuropeptide in the brain and promotes the mother's protective behavior toward her young. In humans, oxytocin has been shown to impact empathy, generosity, sexuality, conjugal and social bonding, and stress reactivity (MacDonald and MacDonald, 2010). Despite this, it is not easy to determine the precise causal relationship between OT and the attachment system. Current evidence suggests a reciprocal and two-way relationship.

OT is synthesized in the hypothalamus, and OT signaling has been extensively linked to pro-social and attachment behavior (Galbally et al., 2011). In monogamous prairie voles, the oxytocinergic system promotes resilience to the effects of neonatal isolation on adult social attachment (Barrett et al., 2015). Early life stress and attachment profile can have epigenetic implications for the expression of the oxytocin receptor gene (OXTR) (Feldman et al., 2016; Pearce et al., 2017; Ein-Dor et al., 2018). Increased DNA methylation of the structural gene for oxytocin (OXT) has also been linked to higher levels of attachment insecurity in adults (Haas et al., 2016). Strathearn et al. (2009) and Pierrehumbert et al. (2012) report differential oxytocin responses to stressors based on differences in adult attachment styles, which are laid down chiefly during early childhood (see section Attachment and Social Functioning).

Many factors contribute to individual variations in the response to stressful experiences. Pierrehumbert et al. (2012) evaluated stress response patterns based on adult attachment style in a community sample as well as in subjects who had been exposed to traumatic events such as abuse or life-threatening diseases during childhood and/or adolescence. Subjects with an avoidant attachment style reported moderate subjective stress, high HPA response, and moderate oxytocin levels. Subjects with an anxious attachment style had moderate levels of subjective stress, HPA response, and relatively low levels of oxytocin. Finally, subjects with a disorganized attachment style reported high subjective stress; they had a suppressed HPA response and moderate levels of oxytocin. These data support the notion that attachment styles may affect stress responses and suggest a specific role for oxytocin in the attachment and stress systems.

However, it is unlikely that attachment is driven by the signaling of a single neuropeptide. The opioidergic, dopaminergic, prolactinergic, and vasopressinergic systems are all closely linked to the oxytocinergic system, and these systems are likely to drive attachment behavior in concert (Insel, 1997; Machin and Dunbar, 2011; Pearce et al., 2017). As put forward by the brain opioid theory of social attachment (Panksepp et al., 1978, 1980; Nelson and Panksepp, 1998; Machin and Dunbar, 2011; Loseth et al., 2014; Inagaki, 2018), the signaling of endogenous opioids, and specifically μ-opioids in the brain is critical both to feelings of social connection and social loss, i.e., separation distress. It is important to note that “social attachment” in terms of this theory refers to social bonds generally, and not specifically those formed during early interactions with the caregiver. Indeed, the developmental link between early attachment and the opioidergic system needs further investigation. However, differences in adult attachment style have been linked to differences in the expression of μ-opiod receptor genes (Troisi et al., 2012; Pearce et al., 2017) as well as to differences in the availability of μ-opioid receptors in the brain (Nummenmaa et al., 2015). In their work on the link between early attachment, separation distress and depression (see section Effects of Social Relations on Cognitive Decline), Watt and Panksepp (2009) also emphasize the importance of the opioidergic system, as well as that of the oxytocinergic and other neurotransmitter systems, the HPA axis, and immune responses. As social bonds can play an important role in maintaining cognitive abilities in old age, the potential impact of early attachment on the signaling of socially relevant neuropeptides provides another example of how early secure attachment could protect against cognitive decline later in life.

#### Neuroinflammation

It is also important to consider the role that neuroinflammation could play in mediating the impact of early attachment on cognitive capacity later in life. Neuroinflammation is thought to play an important role in Alzheimer's disease pathology (see Heneka et al., 2015 for a review), as well as in the pathology of other dementias (see Pasqualetti et al., 2015). Evidence suggests that early life stress can have lifelong consequences for susceptibility to neuroinflammation in rodents (Ganguly and Brenhouse, 2015; Roque et al., 2016; Hoeijmakers et al., 2017). Social stress and insecure early attachment have also been associated with inflammatory responses in humans (e.g., Gouin et al., 2009; Slavich et al., 2010; see Ehrlich, 2019, for a recent review of the links between adult attachment and psychoneuroimmunology, with a specific focus on inflammatory responses). Neuroinflammation is likely to constitute yet another mechanism through which stress can lead to cognitive decline (Hoeijmakers et al., 2018). Indeed, aside from the potential impact of early life stress on neuroinflammation, neuroinflammation may also mediate the link between later life stress and depression on the one hand, and cognitive decline and dementia on the other hand (Leonard, 2007; García-Bueno et al., 2008; Slavich and Irwin, 2014; Miller and Raison, 2016; Santos et al., 2016; Bisht et al., 2018; Justice, 2018).

### Implications for Dementia Related Pathologies

Crucially, many of the brain structures and systems that are impacted by early adverse attachment experience and early life stress are also implicated in dementia-related neuropathology. For example, the two neuropathological hallmarks of Alzheimer's disease, neurofibrillary tangles (NFT) and amyloid containing senile plaques (SP) (alongside synaptic and neuronal loss), typically emerge in medio-temporal lobe areas such as the hippocampus, entorhinal cortex and amygdala, before spreading to areas of the neocortex (von Gunten et al., 2006; Giannakopoulos et al., 2009; Perl, 2010; Sperling et al., 2011; Nelson et al., 2012; Yang et al., 2012). Dysregulation in the HPA axis has been observed both in Alzheimer's disease and other dementias and has been linked to worsening cognition. Thus, HPA dysregulation constitutes one likely mechanism through which stress can lead to cognitive decline and possibly dementia (Lupien et al., 1998; Magri et al., 2006; Gil-Bea et al., 2010; Gupta and Morley, 2014; Popp et al., 2015; Pietrzak et al., 2017; Caruso et al., 2018). As brought forward above, neuroinflammation and changes in the signaling of neuropeptides and insulin-like growth factor are also likely to play a role in neurodegeneration and cognitive decline. Finally, although PFC damage can be observed in early stages of Alzheimer's disease (von Gunten et al., 2005, 2006), it may be more common in other types of dementia, such as frontotemporal dementia and vascular dementia (e.g., McPherson and Cummings, 1996; Rosen et al., 2002; Neary et al., 2005; Korczyn et al., 2012).

In animal models, current work is starting to link early life stress to the development of specific dementia-related pathologies more directly. For example, in mouse models of Alzheimer's disease, early life stress and maternal separation have been linked to increased amyloid accumulation in the hippocampus and to cognitive deficits (Hoeijmakers et al., 2017; Hui et al., 2017), whereas increased maternal care has been linked to delayed amyloid accumulation and delayed cognitive decline (Lesuis et al., 2017). Recently, Hoeijmakers et al. (2018) reviewed the evidence linking early life stress to enhanced risk for cognitive decline and Alzheimer's disease in rodent models.

### Attachment and the Exploration System

The evidence outlined above points to a clear influence of early attachment experience on neurobiological development, with consequences for cognitive and social functioning across the lifespan. Furthermore, links between the attachment and exploration systems may promote cognitive development. Although the attachment and exploration systems are distinct, they are intrinsically linked, as, in addition to addressing needs of proximity and protection, attachment bonds also promote exploration behavior (Ainsworth and Wittig, 1969; Bowlby, 1969, 1980; Ainsworth et al., 1978). Such exploration is driven by what Panksepp calls the SEEKING system, which essentially compels an individual to explore the environment in response to appetitive needs (Ellis and Solms, 2017). Such exploration includes investigation of and engagement with the environment (Panksepp, 1998; Bergin and Bergin, 2009).

The level of attachment security is reflected in the balance between comfort seeking behaviors and the drive to explore the environment (Ainsworth, 1985; Weinfield et al., 1999). When the child feels sufficient confidence in their relationship with the caregiver, as well as confidence in the availability of the caregiver in case of need, this will allow the activation of the exploration system (Grossmann et al., 2008; Weinfield et al., 2008). When facing a threat, discomfort or challenging situation, children with a secure attachment profile have the ability to search for support and comfort from their caregiver. After being reassured and comforted, they may return to their exploratory activities. As they have the ability to internalize a representation of a positive and reliable caregiver, secure children tend to invest themselves in more challenging investigations, which may in turn induce greater cognitive stimulation (Bretherton, 1985; Bus et al., 1995).

Insecure children do not demonstrate the same balance between exploration and attachment. Anxious children maintain attachment behaviors even in the absence of threatening or harmful situations. As a result, they are unable to invest fully in the exploration of their environment (Ainsworth and Bell, 1970). On the contrary, when faced with threatening or stressful situations, avoidant infants suppress their attachment needs and appear to be able to maintain their exploratory activities without expressing the need for support. These children will therefore not experience the same beneficial interactions with the caregiver as secure children would (O'Connor and McCartney, 2007).

An increased ability to interact with the environment and social world will promote cognitive skills and favor the development of neural networks and cognitive functions central to self-regulation (Bernier et al., 2010; Stievenart et al., 2011). This is in line with animal research that has shown that frequent and diversified activity increases the number of neurons and synapses and positively influences brain and cognitive reserves (Churchill et al., 2002). Indeed, some studies have demonstrated that exploration mediates the link between attachment and later cognitive skills (O'Connor and McCartney, 2007; West et al., 2013; McCormick et al., 2016). O'Connor and McCartney (2007), observed that the effect of insecure attachment on cognitive skills in first grade children is attributable to various factors. Specifically, insecure children showed a low level of commitment to tasks, demonstrated reduced exploration, received poor quality maternal assistance, maintained poor quality relationships with teachers, and demonstrated low-level communication and attention skills, which were in turn associated with lower levels of cognitive abilities.

Therefore, in addition to the neurodevelopmental impact of early attachment, we have discussed how the attachment system may promote social interaction and cognitive development. Taken together, these processes could favor the development of brain and cognitive reserve and, thus, protect against later cognitive decline or dementia.

## Protective Aspects of Brain and Cognitive Reserve

Aging may be associated with changes in cognitive performance as well as neurological changes on the chemical, structural and functional level. The concepts of brain and cognitive reserve (BCR) have been put forward as explanations for the frequent miss-match between the severity of neurodegeneration and the severity of its clinical manifestation. Inter-individual differences in available BCR may explain differences in the extent to which cognitive performance is preserved following neurodegeneration (Stern, 2002, 2009). BCR should protect both against the adverse consequences of decline due to normal aging, as well as against damage due to degenerative diseases or other pathological processes or events. We hypothesize that one of the mechanisms whereby early social interactions may promote the maintenance of cognitive abilities in later life is by contributing to the development of BCR.

The terms cognitive and brain reserve have been used somewhat interchangeably in the literature (Roe et al., 2007; Nithianantharajah and Hannan, 2011). Initially, the term brain reserve was used to describe inter-individual differences in certain quantitative properties of the brain, which might protect against the clinical manifestations of brain damage or degeneration (Satz, 1993; Stern, 2012). For example, individuals with larger brain size, or a higher number of neurons and synapses, may be able to sustain more extensive neurodegeneration before clinical manifestations emerge than individuals with lower levels of such “brain reserve” (Katzman et al., 1988; Katzman, 1993; Schofield et al., 1997; Van Loenhoud et al., 2018). According to this model, reserve was originally conceived as passive and predefined, and clinical symptoms should be observed once pathological alterations surpass a certain fixed threshold (Satz, 1993). By contrast, the concept of cognitive reserve was put forward to describe processes through which an individual might actively counteract or compensate for neuropathology, through the activation of cognitive systems and neural networks (Stern, 2002, 2009). Consequently, individuals with higher levels of education, intelligence, or occupational attainment may be better equipped to resist the clinical impact of brain damage, due to more efficient processing or the ability to recruit new neural networks when performing complex tasks (Stern, 2009). As such, cognitive reserve enhances the ability to make use of damaged resources in order to perform tasks successfully.

Although conceptually distinguishable, brain reserve and cognitive reserve are related one to the other (Nithianantharajah and Hannan, 2011; Stern, 2012). For example, cognitive reserve built up by education or general intelligence may be related to aspects of brain structure, such as increased synaptic density (Katzman, 1993). Brain reserve may not be as static as was originally proposed due, for example, to the potential for adult neurogenesis or enhanced neural plasticity as a result of upregulated BDNF (Stern, 2012; Van Loenhoud et al., 2018). For these reasons, we follow Nithianantharajah and Hannan (2009) in using the term BCR to refer to these ideas collectively.

Various lifestyle and environmental factors have been associated with BCR. Epidemiological studies have provided substantial evidence that factors such as linguistic ability in young adulthood, education (e.g., more years of formal schooling), intellectually stimulating work and engaging in leisure activities can slow cognitive decline and delay the onset of dementia (e.g., Snowdon et al., 1996; Fratiglioni and Wang, 2007; Sharp and Gatz, 2011; Pool et al., 2016; Soldan et al., 2017; Wang et al., 2017; Groot et al., 2018). A number of studies have focused specifically on the impact of early childhood education and socioeconomic environment on BCR. They suggest that this period may be critical for reducing the rate of cognitive decline and the risk of dementia later in life (Stern et al., 1994; De Ronchi et al., 1998; Moceri et al., 2000, 2001; Ravona-Springer et al., 2012; Dekhtyar et al., 2015; Zahodne et al., 2015). Early education may be important for BCR as it occurs during critical neurodevelopmental windows (Zahodne et al., 2015).

Recently, Lesuis et al. (2018) have argued for an important link between the early life environment and BCR. After reviewing evidence from rodent studies, they suggest that early life experiences may influence BCR, cognitive decline and the development of Alzheimer's pathology through a variety of mechanisms. These mechanisms may include altering dendritic and synaptic complexity and programming the HPA axis and the neuroinflammatory response. Correspondingly, we would like to argue that many of the mechanisms underlying the positive influence of early attachment on cognitive and neurobiological development (see section Influence of Early Attachment on Neurobiological and Cognitive Development) could protect against cognitive decline and dementia by acting on BCR. The socio-affective mechanisms whereby early attachment may protect against later cognitive decline (outlined in the section on Attachment, Social Factors, Cognitive Decline and Dementia) could be interpreted similarly. Indeed, a number of previous authors have highlighted the potential link between social factors such as network size, social support and social satisfaction with BCR (Glymour et al., 2008; Amieva et al., 2010; Stoykova et al., 2011). However, the empirical evidence remains limited, and further research is needed to test if, and how, early attachment contributes to BCR.

## Conclusion and Future Perspectives

**Figure 1:**
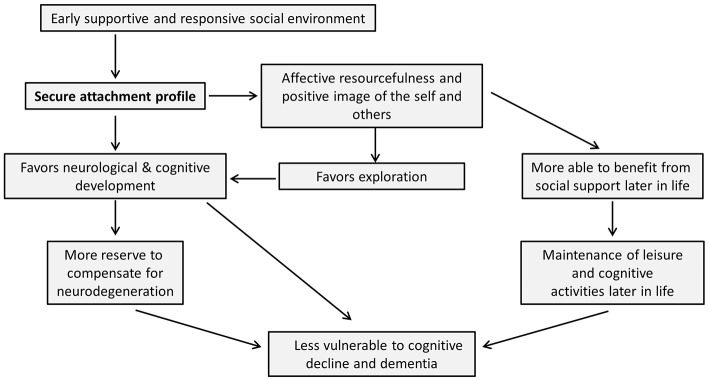
Schematic summary of article. In this figure, we summarize the arguments put forward in this review. It is important to note that, while the arrows in this figure are unidirectional (thereby keeping with the main thrust of the article), it is likely that the influence between many of the nodes in this scheme could in fact run in both directions. However, we have chosen one-directional arrows as we believe these most accurately demonstrate the arguments brought forward in this review, which specifically emphasize the proposed impact of early secure attachment on later cognitive decline and dementia.

Although developmental psychology has traditionally focused on the progression from childhood into young adulthood, this review draws attention to the potentially long-lasting effects of early life experiences and early developmental processes into old age. For a schematic summary of the arguments put forward in this review, please see Figure 1. In line with Bowlby's words “from the cradle to the grave,” we have offered evidence of the continuation of the effects of primary attachment relations from early childhood to old age. The quality of early life interactions influences neurobiological, cognitive, affective and social development and may thereby protect against later cognitive decline. On the one hand, early attachment experiences could influence the will to maintain social engagements and relationships later in life, as well as the perceived quality of social support. On the other hand, attachment experiences may influence—through their influence on neurobiological development and cognitive functioning—the development or availability of brain and cognitive reserve. Furthermore, affective and social consequences of attachment experiences may themselves be able to foster the successful functioning and maintenance of these reserves. Perhaps, alongside the notions of brain and cognitive reserve, we may want to introduce the idea of “affective reserve,” which would explain how favorable affective resources might protect against cognitive decline.

Taken together, BCR and affective reserve could explain the differences observed in the way elderly people cope with age-related changes. In a similar vein, an increasing interest in the study of resilience in elderly people is currently emerging (MacLeod et al., 2016; Arenaza-Urquijo and Vemuri, 2018). Resilience refers to the capacity for positive adaptation in the face of life adversity, trauma or significant sources of stress. In older adults, resilience is mostly studied in the context of well-being, successful aging and preserved functioning (Ong et al., 2009). In line with the arguments brought forward in this review, early life experiences may favor the development of these adaptive processes, which may in turn help elderly people to cope successfully with significant somatic, psychological and environmental changes (Lesuis et al., 2018). Thus, resilience could be seen as similar to our notion of “affective reserve.” A deeper understanding of affective and social contributors, and the manner in which they interact with more traditional ideas of BCR, could be crucial to elucidating how cognitive functioning may be maintained with age (Bartrés-Faz et al., 2018).

It is up to future work to test these ideas. The link between early attachment, BCR and affective reserve, and eventual cognitive decline and dementia needs to be investigated more directly and in greater detail. If such a link is confirmed, it may well-prove useful in the quest for early identification of individuals at risk of developing dementia and in suggesting new avenues for interventions.

## Author Contributions

EW, YB, and AD equally contributed to the writing of the manuscript. All authors contributed to the conception and design of the review and/or revisions, manuscript revision, read and approved the submitted version.

### Conflict of Interest Statement

The authors declare that the research was conducted in the absence of any commercial or financial relationships that could be construed as a potential conflict of interest.

## References

[B1] Ainsworth M. D. (1985). Patterns of infant-mother attachments: antecedents and effects on development. Bull. N. Y. Acad. Med. 61, 771–791. 3864510 PMC1911899

[B2] Ainsworth M. D. S. (1979). Attachment as related to mother-infant interaction. Adv. Study Behav. 9, 1–51. 10.1016/S0065-3454(08)60032-7

[B3] Ainsworth M. I. S. Bell S. (1970). Attachment, exploration, and separation: illustrated by the behavior of one-year-olds in a strange situation. Child Dev. 41, 49–67. 10.2307/1127388 5490680

[B4] Ainsworth M. I. S. Blehar M. C. Waters E. Wall S. (1978). Patterns of Attachment: A Psychological Study of the Strange Situation. Hillsdale, NJ: Erlbaum.

[B5] Ainsworth M. I. S. Wittig B. A. (1969). Attachment and the exploratory behaviour of one-year-olds in a strange situation, in Determinants of Infant Behaviour, Vol. 4, ed Foss B. M. (London: Methuen, 113–136.

[B6] Akdogan R. (2017). A model proposal on the relationships between loneli ness, insecure attachment, and inferiority feelings. Personality Individual Differences 111, 19–24. 10.1016/j.paid.2017.01.048

[B7] Aleman A. Torres-Aleman I. (2009). Circulating insulin-like growth factor I and cognitive function: neuromodulation throughout the lifespan. Progr. Neurobiol. 89, 256–265. 10.1016/j.pneurobio.2009.07.008 19665513

[B8] Allis C. D. Jenuwein T. (2016). The molecular hallmarks of epigenetic control. Nat. Rev. Genet. 17, 487–500. 10.1038/nrg.2016.59 27346641

[B9] Amieva H. Stoykova R. Matharan F. Helmer C. Antonucci T. C. Dartigues J.-F. (2010). What aspects of social network are protective for dementia? not the quantity but the quality of social interactions is protective up to 15 years later. Psychosom. Med. 72, 905–911. 10.1097/PSY.0b013e3181f5e121 20807876

[B10] Antonucci T. C. (2001). Social relations: an examination of social networks, social support, and sense of control, in Handbook of the Psychology of Aging, 5th Edn., ed Birren J. E. (San Diego, CA: Academic Press, 427–453.

[B11] Arenaza-Urquijo E. M. Vemuri P. (2018). Resistance vs resilience to Alzheimer disease: clarifying terminology for preclinical studies. Neurology 90, 695–703. 10.1212/WNL.0000000000005303 29592885 PMC5894932

[B12] Ashpole N. M. Sanders J. E. Hodges E. L. Yan H. Sonntag W. E. (2015). Growth hormone, insulin-like growth factor-1 and the aging brain. Exp. Gerontol. 68, 76–81. 10.1016/j.exger.2014.10.002 25300732 PMC4388761

[B13] Ayalon L. (2016). Profiles of loneliness in the caregiving unit. Gerontologist 56, 201–214. 10.1093/geront/gnu046 24840915

[B14] Baldwin M. W. Keelan J. P. R. Fehr B. Enns V. Koh-Rangarajoo E. (1996). Social-cognitive conceptualization of attachment working models: availability and accessibility effects. J. Personality Soc. Psychol. 71, 94–109. 10.1037/0022-3514.71.1.94

[B15] Bale T. L. Baram T. Z. Brown A. S. Goldstein J. M. Insel T. R. McCarthy M. M.. (2010). Early life programming and neurodevelopmental disorders. Biol. Psychiatry 68, 314–319. 10.1016/j.biopsych.2010.05.028 20674602 PMC3168778

[B16] Bale T. L. Vale W. W. (2004). CRF and CRF receptors: role in stress responsivity and other behaviors. Annu. Rev. Pharmacol. Toxicol. 44, 525–527. 10.1146/annurev.pharmtox.44.101802.121410 14744257

[B17] Barrett C. E. Arambula S. E. Young L. J. (2015). The oxytocin system promotes resilience to the effects of neonatal isolation on adult social attachment in female prairie voles. Transl. Psychiatry 5:e606. 10.1038/tp.2015.73 26196439 PMC5068726

[B18] Bartholomew K. Horowitz L. M. (1991). Attachment styles among young adults: a test of a four-category model. J. Personality Soc. Psychol. 61:226. 10.1037//0022-3514.61.2.226 1920064

[B19] Bartrés-Faz D. Cattaneo G. Solana J. Tormos J. M. Pascual-Leone A. (2018). Meaning in life: resilience beyond reserve. Alzheimer Res. Ther. 10, 1–10. 10.1186/s13195-018-0381-z 29793549 PMC5968537

[B20] Bassuk S. S. Glass T. A. Berkman L. F. (1999). Social disengagement and incident cognitive decline in community-dwelling elderly persons. Ann. Intern. Med. 131, 165–173. 10.7326/0003-4819-131-3-199908030-00002 10428732

[B21] Beaudreau S. A. O'Hara R. (2008). Late-life anxiety and cognitive impairment: a review. Am. J. Geriatr. Psychiatry 16, 790–803. 10.1097/JGP.0b013e31817945c3 18827225

[B22] Beland F. Zunzunegui M. Alvarado B. Otero A. del Ser T. (2005). Trajectories of cognitive decline and social relations. J. Geronotol. Psychol. Sci. 60B, 320–330. 10.1093/geronb/60.6.P320 16260706

[B23] Belsky J. de Haan M. (2011). Annual research review: parenting and children's brain development: the end of the beginning. J. Child Psychol. Psychiatry 52, 409–428. 10.1111/j.1469-7610.2010.02281.x 20626527

[B24] Bennett D. A. Schneider J. A. Tang Y. Arnold S. E. Wilson R. S. (2006). The effect of social networks on the relation between Alzheimer's disease pathology and level of cognitive function in old people: a longitudinal cohort study. Lancet Neurol. 5, 406–412. 10.1016/S1474-4422(06)70417-3 16632311

[B25] Berant E. Mikulincer M. Florian V. (2001). The association of mothers' attachment style and their psychological reactions to the diagnosis of Infant's Congenital Heart Disease. J. Soc. Clin. Psychol. 20, 208–232. 10.1521/jscp.20.2.208.22264

[B26] Bergin C. Bergin D. (2009). Attachment in the classroom. Edu. Psychol. Rev. 21, 141–170. 10.1007/s10648-009-9104-0

[B27] Bernardon S. Babb K. A. Hakim-Larson J. Gragg M. (2011). Loneliness, attachment, and the perception and use of social support in university students. Can. J. Behav. Sci. 43, 40–51. 10.1037/a0021199

[B28] Bernier A. Carlson S. M. Deschênes M. Matte-Gagn,é C. (2012). Social factors in the development of early executive functioning: a closer look at the caregiving environment. Dev. Sci. 15, 12–24. 10.1111/j.1467-7687.2011.01093.x 22251288

[B29] Bernier A. Carlson S. M. Whipple N. (2010). From external regulation to self-regulation: early parenting precursors of young children's executive functioning. Child Dev. 81, 326–339. 10.1111/j.1467-8624.2009.01397.x 20331670

[B30] Bibok M. B. Carpendale J. I. Müller U. (2009). Parental scaffolding and the development of executive function. New Directions Child Adolesc. Dev. 2009, 17–34. 10.1002/cd.233 19306272

[B31] Bisht K. Sharma K. Tremblay M. È. (2018). Chronic stress as a risk factor for Alzheimer's disease: roles of microglia-mediated synaptic remodeling, inflammation, and oxidative stress. Neurobiol. Stress 9, 9–21. 10.1016/j.ynstr.2018.05.003 29992181 PMC6035903

[B32] Bock J. Gruss M. Becker S. Braun K. (2005). Experience-induced changes of dendritic spine densities in the prefrontal and sensory cortex: correlation with developmental time windows. Cereb. Cortex 15, 802–808. 10.1093/cercor/bhh181 15371297

[B33] Bosmans G. Young J. F. Hankin B. L. (2018). NR3C1 methylation as a moderator of the effects of maternal support and stress on insecure attachment development. Dev. Psychol. 54, 29–38. 10.1037/dev0000422 29058930 PMC5750115

[B34] Bowlby J. (1958). The nature of the child's tie to his mother. Int. J. Psychoanal. 39, 350–373. 13610508

[B35] Bowlby J. (1969). Attachment and Loss. Vol. 1: Attachment. New York, NY: Basic Books.

[B36] Bowlby J. (1980). Attachment and Loss. Vol. 3: Loss, Sadness and Depression. New York, NY: Basic Books.

[B37] Bowlby J. (1982). Attachment and loss: retrospect and prospect. Am. J. Orthopsychiatr. 52, 664–678. 10.1111/j.1939-0025.1982.tb01456.x 7148988

[B38] Bowlby J. (1988). A Secure Base: Parent-child Attachment and Healthy Human Development. New York, NY: Basic Books.

[B39] Bradley J. M. Cafferty T. P. (2001). Attachment among older adults: current issues and directions for future research. Attachment Human Dev. 2, 200–221. 10.1080/14616730110058016 11708737

[B40] Brennan K. A. Morris K. A. (1997). Attachment styles, self-esteem, and patterns of feedback seeking from romantic partners. Personality Soc. Psychol. Bull. 23, 23–31. 10.1177/0146167297231003

[B41] Bretherton I. (1985). Attachment theory: retrospect and prospect. Monographs Soc. Res. Child Dev. 50, 3–35. 10.2307/3333824

[B42] Bretherton I. (1995). A communication perspective on attachment relationships and internal working models. Monographs Soc. Res. Child Dev. 60, 310–329. 10.2307/1166187

[B43] Bretherton I. Munholland K. A. (1999). Internal working models in attachment relationships: a construct revisited, in Handbook of Attachment: Theory, Research, and Clinical Applications, eds Cassidy J. Shaver P. R. (New York, NY: The Guilford Press, 89–111.

[B44] Brummelte S. (2017). Introduction: early adversity and brain development. Neuroscience 342, 1–3. 10.1016/j.neuroscience.2016.09.041 27670901

[B45] Brunson K. L. Kramar E. Lin B. Chen Y. Colgin L. L. Yanagihara T. K.. (2005). Mechanisms of late-onset cognitive decline after early-life stress. J. Neurosci. 25, 9328–9338. 10.1523/JNEUROSCI.2281-05.2005 16221841 PMC3100717

[B46] Burg M. M. Barefoot J. Berkman L. Catellier D. J. Czajkowski S. Saab P.. (2005). Low perceived social support and post-myocardial infarction prognosis in the enhancing recovery in coronary heart disease clinical trial: the effects of treatment. Psychosomat. Med. 67, 879–888. 10.1097/01.psy.0000188480.61949.8c 16314592

[B47] Burgdorf J. Kroes R. A. Beinfeld M. C. Panksepp J. Moskal J. R. (2010). Uncovering the molecular basis of positive affect using rough-and-tumble play in rats: a role for insulin-like growth factor 1. Neuroscience 168, 769–777. 10.1016/j.neuroscience.2010.03.045 20350589

[B48] Burghy C. A. Stodola D. E. Ruttle P. L. Molloy E. K. Armstrong J. M. Oler J. A.. (2012). Developmental pathways to amygdala-prefrontal function and internalizing symptoms in adolescence. Nat. Neurosci. 15, 1736–1741. 10.1038/nn.3257 23143517 PMC3509229

[B49] Bus A. G. van IJzendoorn M. H. Pellegrini A. D. (1995). Joint book reading makes for success in learning to read: a meta-analysis on intergenerational transmission of literacy. Rev. Edu. Res. 65, 1–21. 10.3102/00346543065001001

[B50] Buss C. Lord C. Wadiwalla M. Hellhammer D. H. Lupien S. J. Meaney M. J. (2007). Maternal care modulates the relationship between prenatal risk and hippocampal volume in women but not in men. J. Neurosci. 27, 2592–2595. 10.1523/JNEUROSCI.3252-06.2007 17344396 PMC6672503

[B51] Byers A. L. Yaffe K. (2011). Depression and risk of developing dementia. Nat. Rev. Neurol. 7, 323–331. 10.1038/nrneurol.2011.60 21537355 PMC3327554

[B52] Cacioppo J. T. Hughes M. E. Waite L. J. Hawkley L. C. Thisted R. A. (2006). Loneliness as a specific risk factor for depressive symptoms: cross-sectional and longitudinal analyses. Psychol. Aging 21, 140–151. 10.1037/0882-7974.21.1.140 16594799

[B53] Caldji C. Diorio J. Meaney M. J. (2000). Variations in maternal care in infancy regulate the development of stress reactivity. Biol. Psychiatry 48, 1164–1174. 10.1016/S0006-3223(00)01084-2 11137058

[B54] Caldji C. Tannenbaum B. Sharma S. Francis D. Plotsky P. M. Meaney M. J. (1998). Maternal care during infancy regulates the development of neural systems mediating the expression of fearfulness in the rat. Proc. Natl. Acad. Sci. U.S.A. 95, 5335–5340. 10.1073/pnas.95.9.5335 9560276 PMC20261

[B55] Calkins S. D. Leerkes E. M. (2004). Early attachment processes and the development of emotional self-regulation, in Handbook of Self-regulation: Research, Theory, and Applications, eds Vohs K. D. Baumeister R. F. (New York, NY: Guilford Press, 324–339.

[B56] Callaghan B. L. Tottenham N. (2015). The neuro-environmental loop of plasticity: a cross-species analysis of parental effects on emotion circuitry development following typical and adverse caregiving. Neuropsychopharmacology 41, 1–14. 10.1038/npp.2015.204 26194419 PMC4677125

[B57] Carlson E. A. Egeland B. (2004). The construction of experience: a longitudinal study of representation and behavior. Child Dev. 75, 66–83. 10.1111/j.1467-8624.2004.00654.x 15015675

[B58] Carnelley K. B. Pietromonaco P. R. Jaffe K. (1994). Depression, working models of others, and relationship functioning. J. Personality Soc. Psychol. 66, 127–140. 10.1037/0022-3514.66.1.127 8126643

[B59] Caruso A. Nicoletti F. Mango D. Saidi A. Orlando R. Scaccianoce S. (2018). Stress as risk factor for Alzheimer's disease. Pharmacol. Res. 132, 130–134. 10.1016/j.phrs.2018.04.017 29689315

[B60] Carver C. S. (1997). Adult attachment and personality: converging evidence and a new measure. Personality Soc. Psychol. Bull. 23, 865–883. 10.1177/0146167297238007

[B61] Cassidy J. (1994). Emotion regulation: influences of attachment relationships. Monographs Soc. Res. Child Dev. 59, 228–249. 10.2307/1166148 7984163

[B62] Cassidy J. (2000). Adult romantic attachments: a developmental perspective on individual differences. Rev. General Psychol. 4:111 10.1037//1089-2680.4.2.111

[B63] Cassidy J. Berlin L. J. (1994). The insecure/ambivalent pattern of attachment: theory and research. Child Dev. 65, 971–991. 10.2307/1131298 7956474

[B64] Cecil C. A. M. Viding E. Fearon P. Glaser D. McCrory E. J. (2017). Disentangling the mental health impact of childhood abuse and neglect. Child Abuse Neglect. 63, 106–119. 10.1016/j.chiabu.2016.11.024 27914236

[B65] Champagne D. L. Bagot R. C. van Hasselt F. Ramakers G. Meaney M. J. de Kloet E. R.. (2008). Maternal care and hippocampal plasticity: evidence for experience-dependent structural plasticity, altered synaptic functioning, and differential responsiveness to glucocorticoids and stress. J. Neurosci. 28, 6037–6045. 10.1523/JNEUROSCI.0526-08.2008 18524909 PMC6670331

[B66] Champagne F. A. (2008). Epigenetic mechanisms and the transgenerational effects of maternal care. Front. Neuroendocrinol. 29, 386–397. 10.1016/j.yfrne.2008.03.003 18462782 PMC2682215

[B67] Champagne F. A. Francis D. D. Mar A. Meaney M. J. (2003). Variations in maternal care in the rat as a mediating influence for the effects of environment on development. Physiol. Behav. 79, 359–371. 10.1016/S0031-9384(03)00149-5 12954431

[B68] Chen Y. Baram T. Z. (2016). Toward understanding how early-life stress reprograms cognitive and emotional brain networks. Neuropsychopharmacology 41, 197–206. 10.1038/npp.2015.181 26105143 PMC4677123

[B69] Chocyk A. Bobula B. Dudys D. Przyborowska A. Majcher-Maslanka I. Hess G.. (2013). Early-life stress affects the structural and functional plasticity of the medial prefrontal cortex in adolescent rats. Eur. J. Neurosci. 38, 2089–2107. 10.1111/ejn.12208 23581639

[B70] Churchill J. D. Galvez R. Colcombre S. J. Swain R. A. Kramer A. F. Greenough W. T. (2002). Exercise, experience and the aging brain. Neurobiol. Aging 23, 941–955. 10.1016/S0197-4580(02)00028-3 12392797

[B71] Clark C. A. Sheffield T. D. Chevalier N. Nelson J. M. Wiebe S. A. Espy K. A. (2013). Charting early trajectories of executive control with the shape school. Dev. Psychol. 49, 1481–1493. 10.1037/a0030578 23106846 PMC10860163

[B72] Collins N. Read S. (1994). Cognitive representations of attachment: the content and function of working models, in Advances in Personal Relationships, Vol. 5, eds Bartholomew K. Perlman D. (London: Jessica Kingsley, 53–90.

[B73] Collins N. L. Feeney B. C. (2000). A safe haven: an attachment theory perspective on support seeking and caregiving in intimate relationships. J. Personality Soc. Psychol. 78, 1053–1073. 10.1037/0022-3514.78.6.1053 10870908

[B74] Collins N. L. Read S. J. (1990). Adult attachment, working models, and relationship quality in dating couples. J. Personality Soc. Psychol. 58, 644–663. 10.1037/0022-3514.58.4.644 14570079

[B75] Coplan J. D. Fathy H. M. Jackowski A. P. Tang C. Y. Perera T. D. Mathew S. J.. (2014). Early life stress and macaque amygdala hypertrophy: preliminary evidence for a role for the serotonin transporter gene. Front. Behav. Neurosci. 8, 1–10. 10.3389/fnbeh.2014.00342 25339875 PMC4186477

[B76] Crooks V. C. Lubben J. Petitti D. B. Little D. Chiu V. (2008). Social network, cognitive function, and dementia incidence among elderly women. Am. J. Public Health 98, 1221–1227. 10.2105/AJPH.2007.115923 18511731 PMC2424087

[B77] Crugnola C. R. Tambelli R. Spinelli M. Gazzotti S. Caprin C. Albizzati A. (2011). Attachment patterns and emotion regulation strategies in the second year. Infant Behav. Dev. 34, 136–151. 10.1016/j.infbeh.2010.11.002 21195479

[B78] Da Silva J. Gonçalves-Pereira M. Xavier M. Mukaetova-Ladinska E. B. (2013). Affective disorders and risk of developing dementia: systematic review. Br. J. Psychiatry 202, 177–186. 10.1192/bjp.bp.111.101931 23457181

[B79] Davidson R. J. McEwen B. S. (2013). Social influences on neuroplasticity: stress and interventions to promote well-being. Nat. Neurosci. 15, 689–695. 10.1038/nn.3093 22534579 PMC3491815

[B80] De Kloet E. R. Joëls M. Holsboer F. (2005). Stress and the brain: from adaptation to disease. Nat. Rev. Neurosci. 6, 463–475. 10.1038/nrn1683 15891777

[B81] De Ronchi D. Fratiglioni L. Rucci P. Paternico A. Graziani S. Dalmonte E. (1998). The effect of education on dementia occurrence in an Italian population with middle to high socioeconomic status. Neurology 50, 1231–1238. 10.1212/WNL.50.5.1231 9595968

[B82] De Ruiter C. van IJzendoorn M. H. (1993). Attachment and cognition: a review of the literature. Int. J. Edu. Res. 18, 525–540.

[B83] Dekhtyar S. Wang H. X. Scott K. Goodman A. Ilona K. Herlitz A. (2015). A life-course study of cognitive reserve in dementia—from childhood to old age. Am. J. Geriatr. Psychiatry 23, 885–896. 10.1016/j.jagp.2015.02.002 25746486

[B84] Demir-Lira Ö. E. Voss J. L. O'Neil J. T. Briggs-Gowan M. J. Wakschlag L. S. Booth J. R. (2016). Early-life stress exposure associated with altered prefrontal resting-state fMRI connectivity in young children. Dev. Cogn. Neurosci. 19, 107–114. 10.1016/j.dcn.2016.02.003 27010576 PMC4912914

[B85] Dennis M. Wakefield P. Molloy C. Andrews H. Friedman T. (2005). Self-harm in older people with depression: comparison of social factors, life events and symptoms. Br. J. Psychiatry 186, 538–539. 10.1192/bjp.186.6.538 15928367

[B86] Derks N. A. Krugers H. J. Hoogenraad C. C. Joëls M. Sarabdjitsingh R. A. (2017). Effects of early life stress on rodent hippocampal synaptic plasticity: a systematic review. Curr. Opin. Behav. Sci. 14, 155–166. 10.1016/j.cobeha.2017.03.005

[B87] Diamond A. (2002). Normal development of prefrontal cortex from birth to young adulthood: cognitive functions, anatomy, and biochemistry, in Principles of Frontal Lobe Function, eds Stuss D. Knight R. (New York, NY: Oxford University Press), 466–503. 10.1093/acprof:oso/9780195134971.003.0029

[B88] Diamond A. (2013). Executive functions. Ann. Rev. Psychol. 64, 135–168. 10.1146/annurev-psych-113011-143750 23020641 PMC4084861

[B89] Diamond A. Barnett W. S. Thomas J. Munro S. (2007). Preschool program improves cognitive control. Science 318, 1387. 10.1126/science.1151148 18048670 PMC2174918

[B90] Dickinson W. J. Potter G. G. Hybels C. F. Mcquoid D. R. Steffens D. C. (2011). Change in stress and social support as predictors of cognitive decline in older adults with and without depression. Int. J. Geriatr. Psychiatry 26, 1267–1274. 10.1002/gps.2676 21370277 PMC3280427

[B91] Doi T. Shimada H. Makizako H. Tsutsumimoto K. Hotta R. Nakakubo S.. (2015). Association of insulin-like growth factor-1 with mild cognitive impairment and slow gait speed. Neurobiol. Aging 36, 942–947. 10.1016/j.neurobiolaging.2014.10.035 25467636

[B92] Donovan N. J. Amariglio R. E. Zoller A. S. Rudel R. K. Gomez-Isla T. Blacker D.. (2014). Subjective cognitive concerns and neuropsychiatric predictors of progression to the early clinical stages of Alzheimer disease. Am. J. Geriatr. Psychiatry 22, 1642–1651. 10.1016/j.jagp.2014.02.007 24698445 PMC4145054

[B93] Donovan N. J. Wu Q. Rentz D. M. Sperling R. A. Marshall G. A. Glymour M. M. (2017). Loneliness, depression and cognitive function in older U.S. adults. Int. J. Geriatr. Psychiatry 32, 564–573. 10.1002/gps.4495 27162047 PMC5102822

[B94] Dotson V. M. Beydoun M. A. Zonderman A. B. (2010). Recurrent depressive symptoms and the incidence of dementia and mild cognitive impairment. Neurology 75, 27–34. 10.1212/WNL.0b013e3181e62124 20603482 PMC2906403

[B95] Dyer A. H. Vahdatpour C. Sanfeliu A. Tropea D. (2016). The role of insulin-like growth factor 1 (IGF-1) in brain development, maturation and neuroplasticity. Neuroscience 325, 89–99. 10.1016/j.neuroscience.2016.03.056 27038749

[B96] Ehrlich K. B. (2019). Attachment and psychoneuroimmunology. Curr. Opin. Psychol. 25, 96–100. 10.1016/j.copsyc.2018.03.012 29631123 PMC9358714

[B97] Eiland L. Ramroop J. Hill M. N. Manley J. McEwen B. S. (2012). Chronic juvenile stress produces corticolimbic dendritic architectural remodeling and modulates emotional behavior in male and female rats. Psychoneuroendocrinology 37, 39–47. 10.1016/j.psyneuen.2011.04.015 21658845 PMC3181388

[B98] Ein-Dor T. Verbeke W. J. M. I. Mokry M. Vrtička P. (2018). Epigenetic modification of the oxytocin and glucocorticoid receptor genes is linked to attachment avoidance in young adults. Attachment Human Dev. 20, 439–454. 10.1080/14616734.2018.1446451 29513137

[B99] Ellis G. Solms M. (2017). Beyond Evolutionary Psychology: How and Why Neuropsychological Modules Arise. Cambridge: Cambridge University Press.

[B100] Ellwardt L. Aartsen M. Deeg D. Steverink N. (2013). Does loneliness mediate the relation between social support and cognitive functioning in later life? Soc. Sci. Med. 98, 116–124. 10.1016/j.socscimed.2013.09.002 24331889

[B101] Fan Y. Pestke K. Feeser M. Aust S. Pruessner J. C. Böker H.. (2015). Amygdala-hippocampal connectivity changes during acute psychosocial stress: joint effect of early life stress and oxytocin. Neuropsychopharmacology 40, 2736–2744. 10.1038/npp.2015.123 25924202 PMC4864649

[B102] Fareri D. S. Tottenham N. (2016). Effects of early life stress on amygdala and striatal development. Dev. Cogn. Neurosci. 19, 233–247. 10.1016/j.dcn.2016.04.005 27174149 PMC4912892

[B103] Feldman R. Monakhov M. Pratt M. Ebstein R. P. (2016). Oxytocin pathway genes: evolutionary ancient system impacting on human affiliation, sociality, and psychopathology. Biol. Psychiatry 79, 174–184. 10.1016/j.biopsych.2015.08.008 26392129

[B104] Feng L. Ng X.-T. Yap P. Li J. Lee T.-S. Håkansson K.. (2014). Marital status and cognitive impairment among community-dwelling chinese older adults: the role of gender and social engagement. Dement. Geriatr. Cogn. Dis. Extra 4, 375–384. 10.1159/000358584 25473404 PMC4241637

[B105] Fiori K. L. Antonucci T. C. Cortina K. S. (2006). Social network typologies and mental health among older adults. J. Gerontol. B Psychol. Sci. Soc. Sci. 61, 25–32. 10.1093/geronb/61.1.P25 16399938

[B106] Fiori K. L. Consedine N. S. Merz E.-M. (2011). Attachment, social network size, and patterns of social exchange in later life. Res. Aging 33, 465–493. 10.1177/0164027511401038

[B107] Fox S. E. Levitt P. Nelson, I. I. I. C. A. (2010). How the timing and quality of early experiences influence the development of brain architecture. Child Dev. 81, 28–40. 10.1111/j.1467-8624.2009.01380.x 20331653 PMC2846084

[B108] Frater J. Lie D. Bartlett P. Mcgrath J. J. (2018). Insulin-like growth factor 1 (IGF-1) as a marker of cognitive decline in normal ageing: a review. Ageing Res. Rev. 42, 14–27. 10.1016/j.arr.2017.12.002 29233786

[B109] Fratiglioni L. Paillard-Borg S. Winblad B. (2004). An active and socially integrated lifestyle in late life might protect against dementia. Lancet Neurol. 3, 343–353. 10.1016/S1474-4422(04)00767-7 15157849

[B110] Fratiglioni L. Wang H. X. (2007). Brain reserve hypothesis in dementia. J Alzheimer Dis. 12, 11–22. 10.3233/JAD-2007-12103 17851191

[B111] Freire A. C. C. Pond,é M. P. Liu A. Caron J. (2017). Anxiety and depression as longitudinal predictors of mild cognitive impairment in older adults. Can. J. Psychiatry 62, 343–350. 10.1177/0706743717699175 28346831 PMC5459229

[B112] Galbally M. Lewis A. J. Ijzendoorn M. Van Permezel M. (2011). The role of oxytocin in mother-infant relations: a systematic review of human studies. Harvard Rev. Psychiatry 19, 1–14. 10.3109/10673229.2011.549771 21250892

[B113] Ganguly P. Brenhouse H. C. (2015). Broken or maladaptive? Altered trajectories in neuroinflammation and behavior after early life adversity. Dev. Cogn. Neurosci. 11, 18–30. 10.1016/j.dcn.2014.07.001 25081071 PMC4476268

[B114] García-Bueno B. Caso J. R. Leza J. C. (2008). Stress as a neuroinflammatory condition in brain: damaging and protective mechanisms. Neurosci. Biobehav. Rev. 32, 1136–1151. 10.1016/j.neubiorev.2008.04.001 18468686

[B115] Geda Y. E. Roberts R. O. Mielke M. M. Knopman D. S. Christianson T. J. Pankratz V. S.. (2014). Baseline neuropsychiatric symptoms and the risk of incident mild cognitive impairment: a population-based study. Am. J. Psychiatry 171, 572–581. 10.1176/appi.ajp.2014.13060821 24700290 PMC4057095

[B116] Gee D. G. Gabard-Durnam L. J. Flannery J. Goff B. Humphreys K. L. Telzer E. H.. (2013). Early developmental emergence of human amygdala-prefrontal connectivity after maternal deprivation. Proc. Natl. Acad. Sci. U.S.A. 110, 15638–15643. 10.1073/pnas.1307893110 24019460 PMC3785723

[B117] Gervai J. (2009). Environmental and genetic influences on early attachment. Child Adolesc. Psychiatry Mental Health 3:25. 10.1186/1753-2000-3-25 19732441 PMC2753321

[B118] Giannakopoulos P. Kövari E. Gold G. Von Gunten A. Hof P. R. Bouras C. (2009). Pathological substrates of cognitive decline in alzheimer's disease. Front. Neurol. Neurosci. 24, 20–29. 10.1159/000197881 19182459

[B119] Gil-Bea F. J. Aisa B. Solomon A. Solas M. del Carmen Mugueta M. Winblad B.. (2010). HPA axis dysregulation associated to apolipoprotein E4 genotype in Alzheimer's disease. J. Alzheimer Dis. 22, 829–838. 10.3233/JAD-2010-100663 20858975

[B120] Glass T. A. Mendes de Leon C. F. Bassuk S. S. Berkman L. F. (2006). Social engagement and depressive symptoms in late life: longitudinal findings. J. Aging Health 18, 604–628. 10.1177/0898264306291017 16835392

[B121] Gluckman P. D. Hanson M. A. Cooper C. Thornburg K. L. (2008). Effect of in utero and early-life conditions on adult health and disease. N. Engl. J. Med. 359, 61–73. 10.1056/NEJMra0708473 18596274 PMC3923653

[B122] Glymour M. M. Weuve J. Fay M. E. Glass T. Berkman L. F. (2008). Social ties and cognitive recovery after stroke: does social integration promote cognitive resilience? Neuroepidemiology 31, 10–20. 10.1159/000136646 18535395 PMC2794277

[B123] Gogtay N. Giedd J. N. Lusk L. Hayashi K. M. Greenstein D. Vaituzis A. C. (2004). Dynamic mapping of human cortical development during childhood through early adulthood. Proc. Natl. Acad. Sci. U.S.A. 101, 8174–8179. 10.1073/pnas.0402680101 15148381 PMC419576

[B124] Gouin J. P. Glaser R. Loving T. J. Malarkey W. B. Stowell J. Houts C.. (2009). Attachment avoidance predicts inflammatory responses to marital conflict. Brain Behav. Immun. 23, 898–904. 10.1016/j.bbi.2008.09.016 18952163 PMC2771542

[B125] Gow A. J. Pattie A. Whiteman M. C. Whalley L. J. Deary I. J. (2007). Social support and successful aging: investigating the relationships between lifetime cognitive change and life satisfaction. J. Individ. Differ. 28, 103–115. 10.1027/1614-0001.28.3.103

[B126] Groh A. M. Roisman G. I. Booth-LaForce C. Fraley R. C. Owen M. T. Cox M. J.. (2014). Stability of attachment security from infancy to late adolescence. Monographs Soc. Res. Child Dev. 79, 51–66. 10.1111/mono.12113 25100089 PMC13245647

[B127] Groot C. van Loenhoud A. C. Barkhof F. van Berckel B. N. M. Koene T. Teunissen C. C.. (2018). Differential effects of cognitive reserve and brain reserve on cognition in Alzheimer disease. Neurology 90, e149–e156. 10.1212/WNL.0000000000004802 29237798

[B128] Gross J. J. John O. P. (2003). Individual differences in two emotion regulation processes: implications for affect, relationships, and well-being. J. Personality Soc. Psychol. 85, 348–362. 10.1037/0022-3514.85.2.348 12916575

[B129] Grossmann K. Grossmann K. E. Kindler H. (2005). Early care and the roots of attachment and partnership representations, in Attachment from Infancy to Adulthood: The Major Longitudinal Studies, eds Grossmann K. E. Grossmann K. Waters E. (New York, NY: Guilford Publications, 98–13.

[B130] Grossmann K. Grossmann K. E. Kindler H. Zimmermann P. (2008). A wider view of attachment and exploration: the influence of mothers and fathers on the development of psychological security from infancy to young adulthood, in Handbook of Attachment: Theory, Research, and Clinical Applications, 2nd Edn eds Cassidy J. Shaver P. R. (New York, NY: Guilford Press, 857–879.

[B131] Gulpers B. Ramakers I. Hamel R. Köhler S. Oude Voshaar R. Verhey F. (2016). Anxiety as a predictor for cognitive decline and dementia: a systematic review and meta-analysis. Am. J. Geriatr. Psychiatry 24, 823–842. 10.1016/j.jagp.2016.05.015 27591161

[B132] Gunnar M. Quevedo K. (2007). The neurobiology of stress and development. Annu. Rev. Psychol. 58, 145–173. 10.1146/annurev.psych.58.110405.085605 16903808

[B133] Gupta D. Morley J. E. (2014). Hypothalamic-pituitary-adrenal (HPA) axis and aging. Comprehens. Physiol. 4, 1495–1510. 10.1002/cphy.c130049 25428852

[B134] Håkansson K. Rovio S. Helkala E. L. Vilska A. R. Winblad B. Soininen H.. (2009). Association between mid-life marital status and cognitive function in later life: population based cohort study. Br. Med. J. 339:b2462. 10.1136/bmj.b2462 19574312 PMC2714683

[B135] Haas B. W. Filkowski M. M. Cochran R. N. Denison L. Ishak A. Nishitani S.. (2016). Epigenetic modification of OXT and human sociability. Proc. Natl. Acad. Sci. U.S.A. 113, E3816–E3823. 10.1073/pnas.1602809113 27325757 PMC4941462

[B136] Hammond S. I. Müller U. Carpendale J. I. Bibok M. B. Liebermann-Finestone D. P. (2012). The effects of parental scaffolding on preschoolers' executive function. Dev. Psychol. 48:271. 10.1037/a0025519 21928877

[B137] Hanson J. L. Nacewicz B. M. Sutterer M. J. Cayo A. A. Schaefer S. M. Rudolph K. D.. (2015). Behavioral problems after early life stress: contributions of the hippocampus and amygdala. Biol. Psychiatry 77, 314–323. 10.1016/j.biopsych.2014.04.020 24993057 PMC4241384

[B138] Hazan C. Shaver P. R. (1990). Love and work: an attachment theoretical perspective. J. Personality Soc. Psychol. 59, 270–280. 10.1037/0022-3514.59.2.270

[B139] Heim C. Binder E. B. (2012). Current research trends in early life stress and depression: review of human studies on sensitive periods, gene-environment interactions, and epigenetics. Exp. Neurol. 233, 102–111. 10.1016/j.expneurol.2011.10.032 22101006

[B140] Heim C. Nemeroff C. B. (1999). The impact of early adverse experiences on brain systems involved in the pathophysiology of anxiety and affective disorders. Biol. Psychiatry 46, 1509–1522. 10.1016/S0006-3223(99)00224-3 10599479

[B141] Helmer C. Damon D. Letenneur L. Fabrigoule C. Barberger-Gateau P. Lafont S.. (1999). Marital status and risk of Alzheimer's disease: a French population-based cohort study. Neurology 53, 1953–1958. 10.1212/WNL.53.9.1953 10599764

[B142] Heneka M. T. Carson M. J. El Khoury J. Landreth G. E. Brosseron F. Feinstein D. L.. (2015). Neuroinflammation in Alzheimer's disease. Lancet Neurol. 14, 388–405. 10.1016/S1474-4422(15)70016-5 25792098 PMC5909703

[B143] Hoeijmakers L. Lesuis S. L. Krugers H. Lucassen P. J. Korosi A. (2018). A preclinical perspective on the enhanced vulnerability to Alzheimer's disease after early-life stress. Neurobiol. Stress 8, 172–185. 10.1016/j.ynstr.2018.02.003 29888312 PMC5991337

[B144] Hoeijmakers L. Ruigrok S. R. Amelianchik A. Ivan D. Dam A. Van Lucassen P. J.. (2017). Early-life stress lastingly alters the neuroinflammatory response to amyloid pathology in an Alzheimer's disease mouse model. Brain Behav. Immun. 63, 160–175. 10.1016/j.bbi.2016.12.023 28027926

[B145] Holwerda T. J. Deeg D. J. Beekman A. T. van Tilburg T. G. Stek M. L. Jonker C. (2014). Feelings of loneliness, but not social isolation, predict dementia onset: results from the Amsterdam Study of the Elderly (AMSTEL). J. Neurol. Neurosurg. Psychiatry 85, 135–142. 10.1136/jnnp-2012-302755 23232034

[B146] Hopkins J. Gouze K. R. Lavigne J. V. (2013). Direct and indirect effects of contextual factors, caregiver depression, and parenting on attachment security in preschoolers. Attachment Human Dev. 15, 155–173. 10.1080/14616734.2013.750702 23383734

[B147] Hostinar C. E. Stellern S. A. Schaefer C. Carlson S. M. Gunnar M. R. (2012). Associations between early life adversity and executive function in children adopted internationally from orphanages. Proc. Natl. Acad. Sci. U.S.A. 109, 17208–17212. 10.1073/pnas.1121246109 23047689 PMC3477377

[B148] Hughes C. (2011). Changes and challenges in 20 years of research into the development of executive functions. Infant Child Dev. 20, 251–271. 10.1002/icd.736

[B149] Hui J. Feng G. Zheng C. Jin H. Jia N. (2017). Maternal separation exacerbates Alzheimer's disease-like behavioral and pathological changes in adult APPswe/PS1dE9 mice. Behav. Brain Res. 318, 18–23. 10.1016/j.bbr.2016.10.030 27771383

[B150] Hulshof H. J. Novati A. Sgoifo A. Luiten P. G. M. Den Boer J. A. Meerlo P. (2011). Maternal separation decreases adult hippocampal cell proliferation and impairs cognitive performance but has little effect on stress sensitivity and anxiety in adult Wistar rats. Behav. Brain Res. 216, 552–560. 10.1016/j.bbr.2010.08.038 20816703

[B151] Inagaki T. K. (2018). Opioids and social connection. Curr. Directions Psychol. Sci. 27, 85–90. 10.1177/0963721417735531

[B152] Insel T. R. (1997). A neurobiological basis of social attachment. Am. J. Psychiatry 1546, 726–735. 10.1176/ajp.154.6.726 9167498

[B153] Ivy A. S. Rex C. S. Chen Y. Dube C. Maras P. M. Grigoriadis D. E.. (2010). Hippocampal dysfunction and cognitive impairments provoked by chronic early-life stress involve excessive activation of CRH receptors. J. Neurosci. 30, 13005–13015. 10.1523/JNEUROSCI.1784-10.2010 20881118 PMC2991143

[B154] James B. D. Wilson R. S. Barnes L. L. Bennett D. A. (2011). Late-life social activity and cognitive decline in old age. J. Int. Neuropsychol. Soc. 17, 998–1005. 10.1017/S1355617711000531 22040898 PMC3206295

[B155] Jardim G. B. G. Novelo M. Spanemberg L. von Gunten A. Nogueira E. Cataldo Neto A. (2018). Influence of childhood abuse, neglect, and dose-effect of maltreatment subtypes on late-life suicide risk beyond depression. Child Abuse Negl. 80, 249–256. 10.1016/j.chiabu.2018.03.029 29631256

[B156] Jardim G. B. G. von Gunten A. Gomes da Silva Filho I. Klarmann Ziegelmann P. Benzano Bumaguin D. Lopes Nogueira E. (2019). Relationship between childhood maltreatment and geriatric depression: the mediator effect of personality traits. Int. Psychogeriatr. 4, 1–9. 10.1017/S1041610219000073 30827285

[B157] Johnson D. E. Gunnar M. R. (2011). Growth failure in institutionalized children. Monographs Soc. Res. Child Dev. 76, 92–126. 10.1111/j.1540-5834.2011.00629.x 25364058 PMC4214390

[B158] Johnson F. K. Delpech J. C. Thompson G. J. Wei L. Hao J. Herman P.. (2018). Amygdala hyper-connectivity in a mouse model of unpredictable early life stress. Transl. Psychiatry 8:49. 10.1038/s41398-018-0092-z 29463821 PMC5820270

[B159] Jorm A. F. (2000). Is depression a risk factor for dementia or cognitive decline? Gerontology 46, 219–227. 10.1159/000022163 10859462

[B160] Justice N. J. (2018). The relationship between stress and Alzheimer's disease. Neurobiol. Stress 8, 127–133. 10.1016/j.ynstr.2018.04.002 29888308 PMC5991350

[B161] Katzman R. (1993). Education and the prevalence of dementia and Alzheimer's disease. Neurology 43, 13–20. 10.1212/WNL.43.1_Part_1.13 8423876

[B162] Katzman R. Terry R. DeTeresa R. Brown T. Davies P. Fuld P.. (1988). Clinical, pathological, and neurochemical changes in dementia: a subgroup with preserved mental status and numerous neocortical plaques. Ann. Neurol. 23, 138–144. 10.1002/ana.410230206 2897823

[B163] Kauppi M. Kawachi I. Batty G. D. Oksanen T. Elovainio M. Pentti J. (2017). Characteristics of social networks and mortality risk: evidence from two prospective cohort studies. Am. J. Epidemiol. 187, 746–753. 10.1093/aje/kwx301 29020140

[B164] Kestly T. A. (2014). The Interpersonal Neurobiology of Play: Brain-Building Interventions for Emotional Well-Being (Norton Series on Interpersonal Neuro-biology). New York, NY: W. W. Norton.

[B165] Kim P. Leckman J. F. Mayes L. C. Newman M. A. Feldman R. Swain J. E. (2010). Perceived quality of maternal care in childhood and structure and function of mothers' brain. Dev. Sci. 13, 662–673. 10.1111/j.1467-7687.2009.00923.x 20590729 PMC3974609

[B166] Kobak R. R. Cole H. E. Ferenz-Gillies R. Fleming W. S. Gamble W. (1993). Attachment and emotion regulation during mother-teen problem solving: a control theory analysis. Child Dev. 231–245. 10.2307/1131448 8436031

[B167] Kolb B. Mychasiuk R. Muhammad A. Li Y. Frost D. O. Gibb R. (2012). Experience and the developing prefrontal cortex. Proc. Natl. Acad. Sci. U.S.A. 109, 17186–17193. 10.1073/pnas.1121251109 23045653 PMC3477383

[B168] Korczyn A. D. Vakhapova V. Grinberg L. T. (2012). Vascular dementia. J. Neurol. Sci. 322, 2–10. 10.1016/j.jns.2012.03.027 22575403 PMC3435447

[B169] Kraemer G. W. (1992). A psychobiological theory of attachment. Behav. Brain Sci. 15, 493–511. 10.1017/S0140525X00069752 24924028

[B170] Kroenke C. H. Kubzansky L. D. Schernhammer E. S. Holmes M. D. Kawachi I. (2006). Social networks, social support, and survival after breast cancer diagnosis. J. Clin. Oncol. 24, 1105–1111. 10.1200/JCO.2005.04.2846 16505430

[B171] Kroenke C. H. Michael Y. L. Poole E. M. Kwan M. L. Nechuta S. Leas E.. (2017). Postdiagnosis social networks and breast cancer mortality in the After Breast Cancer Pooling Project. Cancer 123, 1228-1237. 10.1002/cncr.30440 27943274 PMC5360517

[B172] Kroenke C. H. Quesenberry C. Kwan M. L. Sweeney C. Castillo A. Caan B. J. (2013). Social networks, social support, and burden in relationships, and mortality after breast cancer diagnosis in the Life After Breast Cancer Epidemiology (LACE) study. Breast Cancer Res. Treat. 137, 261–271. 10.1007/s10549-012-2253-8 23143212 PMC4019377

[B173] Kundakovic M. Champagne F. A. (2015). Early-life experience, epigenetics, and the developing brain. Neuropsychopharmacol. Rev. 40, 141–153. 10.1038/npp.2014.140 24917200 PMC4262891

[B174] Larose S. Bernier A. (2001). Social support processes: mediators of attachment state of mind and adjustment in late adolescence. Attachment Human Dev. 3, 96–120. 10.1080/14616730010024762 11708386

[B175] Leonard B. E. (2007). Inflammation, depression and dementia: are they connected? Neurochem. Res. 32, 1749–1756. 10.1007/s11064-007-9385-y 17705097

[B176] Lesuis S. L. Hoeijmakers L. Korosi A. de Rooij S. R. Swaab D. F. Kessels H. W.. (2018). Vulnerability and resilience to Alzheimer's disease: early life conditions modulate neuropathology and determine cognitive reserve. Alzheimer Res. Ther. 10, 1–20. 10.1186/s13195-018-0422-7 30227888 PMC6145191

[B177] Lesuis S. L. Van Hoek B. A. C. E. Lucassen P. J. Krugers H. J. (2017). Early postnatal handling reduces hippocampal amyloid plaque formation and enhances cognitive performance in APPswe/PS1dE9 mice at middle age. Neurobiol. Learning Memory 144, 27–35. 10.1016/j.nlm.2017.05.016 28579367

[B178] Liew J. (2012). Effortful control, executive functions, and education: bringing self-regulatory and social-emotional competencies to the table. Child Dev. Perspect. 6, 105–111. 10.1111/j.1750-8606.2011.00196.x

[B179] Liu D. Diorio J. Day J. C. Francis D. D. Meaney M. J. (2000). Maternal care, hippocampal synaptogenesis and cognitive development in rats. Nat. Neurosci. 3, 799–806. 10.1038/77702 10903573

[B180] Liu D. Tannenbaum B. Baldji C. Francis D. Feedman A. Shakti S.. (1997). Maternal care, hippocampal glucocorticoid receptors, and hypothalamic-pituitary-adrenal responses to stress. Science 277, 1659–1662. 10.1126/science.277.5332.1659 9287218

[B181] Loseth G. E. Ellingsen D. Leknes S. (2014). State-dependent μ -opioid modulation of social motivation. Front. Behav. Neurosci. 8, 1–15. 10.3389/fnbeh.2014.00430 25565999 PMC4264475

[B182] Luby J. L. Belden A. Harms M. P. Tillman R. Barch D. M. (2016). Preschool is a sensitive period for the influence of maternal support on the trajectory of hippocampal development. Proc. Natl. Acad. Sci. U.S.A. 113, 5742–5747. 10.1073/pnas.1601443113 27114522 PMC4878487

[B183] Lupien S. J. de Leon M. de Santi S. Convit A. Tarshish C. Nair N. P.. (1998). Cortisol levels during human aging predict hippocampal atrophy and memory deficits. Nat. Neurosci. 1, 69–73. 10.1038/271 10195112

[B184] Lupien S. J. Parent S. Evans A. C. Tremblay R. E. Zelazo P. D. Corbo V.. (2011). Larger amygdala but no change in hippocampal volume in 10-year-old children exposed to maternal depressive symptomatology since birth. Proc. Natl. Acad. Sci. U.S.A. 108, 14324–14329. 10.1073/pnas.1105371108 21844357 PMC3161565

[B185] Lyons-Ruth K. Pechtel P. Yoon S. A. Anderson C. M. Teicher M. H. (2016). Disorganized attachment in infancy predicts greater amygdala volume in adulthood. Behav. Brain Res. 308, 83–93. 10.1016/j.bbr.2016.03.050 27060720 PMC5017306

[B186] MacDonald K. MacDonald T. M. (2010). The peptide that binds: a systematic review of oxytocin and its prosocial effects in humans. Harvard Rev. Psychiatry 18, 1–21. 10.3109/10673220903523615 20047458

[B187] Machin A. J. Dunbar R. I. M. (2011). The brain opioid theory of social attachment: a review of the evidence. Behaviour 148, 985–1025. 10.1163/000579511X596624

[B188] MacLeod S. Musich S. Hawkins K. Alsgaard K. Wicker E. R. (2016). The impact of resilience among older adults. Geriatr. Nurs. 37, 266–272. 10.1016/j.gerinurse.2016.02.014 27055911

[B189] Magri F. Cravello L. Barili L. Sarra S. Cinchetti W. Salmoiraghi F. (2006). Stress and dementia: the role of the hypothalamic-pituitary-adrenal axis. Aging Clin. Exp. Res. 18, 167–170. 10.1007/BF03327435 16702789

[B190] Main M. (1990). Cross-cultural studies of attachment organization: recent studies, changing methodologies, and the concept of conditional strategies. Human Dev. 33, 48–61. 10.1159/000276502

[B191] Main M. Kaplan N. Cassidy J. (1985). Security in infancy, childhood, and adulthood: a move to the level of representation. Monographs Soc. Res. Child Dev. 50, 66–104. 10.2307/3333827

[B192] Main M. Solomon J. (1986). Discovery of an insecure-disorganized/disoriented attachment pattern, in Affective Development in Infancy, eds Brazelton T. B. Yogman M. W. (Westport, CT: Ablex Publishing, 95–124.

[B193] Malter Cohen M. Jing D. Yang R. R. Tottenham N. Lee F. S. Casey B. J. (2013). Early-life stress has persistent effects on amygdala function and development in mice and humans. Proc. Natl. Acad. Sci. U.S.A. 110, 18274–18278. 10.1073/pnas.1310163110 24145410 PMC3831447

[B194] Matte-Gagné C. Bernier A. (2011). Prospective relations between maternal autonomy support and child executive functioning: investigating the mediating role of child language ability. J. Exp. Child Psychol. 110, 611–625. 10.1016/j.jecp.2011.06.006 21798554

[B195] McClelland M. M. Ponitz C. C. Messersmith E. E. Tominey S. (2010). Self-regulation: integration of cognition and emotion, in The Handbook of Life-span Development, Vol. 1. Cognition, Biology, and Methods, eds Overton W. F. Lerner R. M. (Hoboken, NJ: John Wiley and Sons Inc), 509–553. 10.1002/9780470880166.hlsd001015

[B196] McCormick M. P. O'Connor E. E. Barnes S. P. (2016). Mother– child attachment styles and math and reading skills in middle childhood: the mediating role of children's exploration and engagement. Early Childhood Res. Q. 36, 295–306. 10.1016/j.ecresq.2016.01.011

[B197] McCrory E. J. De Brito S. A. Sebastian C. L. Mechelli A. Bird G. Kelly P. A.. (2011). Heightened neural reactivity to threat in child victims of family violence. Curr. Biol. 21, R947–R948. 10.1016/j.cub.2011.10.015 22153160

[B198] McEwen B. S. Morrison J. H. (2013). The brain on stress: vulnerability and plasticity of the prefrontal cortex over the life course. Neuron 79, 16–29. 10.1016/j.neuron.2013.06.028 23849196 PMC3753223

[B199] McGowan P. O. Sasaki A. D'Alessio A. C. Dymov S. Labont,é B. Szyf M.. (2009). Epigenetic regulation of the glucocorticoid receptor in human brain associates with childhood abuse. Nat. Neurosci. 12, 342–348. 10.1038/nn.2270 19234457 PMC2944040

[B200] McGowan S. (2002). Mental representations in stressful situations: the calming and distressing effects of significant others. J. Exp. Soc. Psychol. 38, 152–161. 10.1006/jesp.2001.1493

[B201] McLaughlin K. A. Peverill M. Gold A. L. Alves S. Sheridan M. A. (2015). Child maltreatment and neural systems underlying emotion regulation. J. Am. Acad. Child Adolesc. Psychiatry 54, 753–762. 10.1016/j.jaac.2015.06.010 26299297 PMC4548288

[B202] McPherson S. E. Cummings J. L. (1996). Neuropsychological aspects of vascular dementia. Brain Cogn. 31, 269–282. 10.1006/brcg.1996.0045 8812007

[B203] Meaney M. J. (2001). Maternal care, gene expression, and the transmission of individual differences in stress reactivity across generations. Annu. Rev. Neurosci. 24, 1161–1192. 10.1146/annurev.neuro.24.1.1161 11520931

[B204] Mehta M. A. Golembo N. I. Nosarti C. Colvert E. Mota A. Williams S. C. R.. (2009). Amygdala, hippocampal and corpus callosum size following severe early institutional deprivation: the English and Romanian Adoptees study pilot. J. Child Psychol. Psychiatry Allied Disciplines 50, 943–951. 10.1111/j.1469-7610.2009.02084.x 19457047

[B205] Mezzacappa E. Buckner J. C. Earls F. (2011). Prenatal cigarette exposure and infant learning stimulation as predictors of cognitive control in childhood. Dev. Sci. 14, 881–891. 10.1111/j.1467-7687.2011.01038.x 21676107 PMC3117204

[B206] Mikulincer M. Dolev T. Shaver P. R. (2004). Attachment-related strategies during thought suppression: ironic rebounds and vulnerable self-representations. J. Personality \Soc. Psychol. 87, 940–956. 10.1037/0022-3514.87.6.940 15598116

[B207] Mikulincer M. Florian V. (1995). Appraisal of and coping with real-life stressful situations: the contributions of attachment styles. Personality Soc. Psychol. Bull. 21, 406–414. 10.1177/0146167295214011

[B208] Mikulincer M. Florian V. (1998). The relationship between adult attachment styles and emotional and cognitive reactions to stressful events, in Attachment Theory and Close Relationships, eds Simpson J. A. Rholes W. S. (New York, NY: Guilford Press, 143–165.

[B209] Mikulincer M. Florian V. (2003). Attachment style and affect regulation: implications for coping with stress and mental health, in Blackwell Handbook of Social Psychology: Interpersonal Processes, eds Fletcher G. J. O. Clark M. S. (New York, NY: Blackwell), 537–557.

[B210] Mikulincer M. Shaver P. R. (2007). Attachment Patterns in Adulthood: Structure, Dynamics and Change. New York, NY: Guilford Press.

[B211] Mikulincer M. Shaver P. R. Pereg D. (2003). Attachment theory and affect regulation: the dynamics, development, and cognitive consequences of attachment-related strategies. Motivat. Emot. 27, 77–102. 10.1023/A:1024515519160

[B212] Miljkovitch R. (2009). Les Fondations du Lien Amoureux. Paris: Presses Universitaires de France.

[B213] Miljkovitch R. Cohin E. (2007). L'attachement dans la relation de couple: une continuité de l'enfance? Dialogue 175, 87–96. 10.3917/dia.175.0087

[B214] Miller A. H. Raison C. L. (2016). The role of inflammation in depression: from evolutionary imperative to modern treatment target. Nat. Rev. Immunol. 16, 22–34. 10.1038/nri.2015.5 26711676 PMC5542678

[B215] Moceri V. M. Kukull W. A. Emanuel I. van Belle G. Larson E. B. (2000). Early-life risk factors and the development of Alzheimer's disease. Neurology 54, 415–420. 10.1212/WNL.54.2.415 10668705

[B216] Moceri V. M. Kukull W. A. Emanuel I. van Belle G. Starr J. R. Schellenberg G. D.. (2001). Using census data and birth certificates to reconstruct the early-life socioeconomic environment and the relation to the development of Alzheimer's disease. Epidemiology 12, 383–389. 10.1097/00001648-200107000-00007 11416775

[B217] Molet J. Maras P. M. Kinney-Lang E. Harris N. G. Rashid F. Ivy A. S.. (2016). MRI uncovers disrupted hippocampal microstructure that underlies memory impairments after early-life adversity. Hippocampus 26, 1618–1632. 10.1002/hipo.22661 27657911 PMC5452614

[B218] Monroy E. Hernandez-Torres E. Flores G. (2010). Maternal separation disrupts dendritic morphology of neurons in prefrontal cortex, hippocampus, and nucleus accumbens in male rat offspring. J. Chem. Neuroanat. 40, 93–101. 10.1016/j.jchemneu.2010.05.005 20553852

[B219] Moss E. St-Laurent D. (2001). Attachment at school age and academic performance. Dev. Psychol. 37:863. 10.1037//0012-1649.37.6.863 11699759

[B220] Neary D. Snowden J. Mann D. (2005). Frontotemporal dementia. Lancet Neurol. 4, 771–780. 10.1016/S1474-4422(05)70223-4 16239184

[B221] Nelson E. E. Panksepp J. (1998). Brain substrates of infant-mother attachment: contributions of opioids, oxytocin, and norepinephrine. Neurosci. Biobehav. Rev. 22, 437–452. 10.1016/S0149-7634(97)00052-3 9579331

[B222] Nelson P. T. Alafuzoff I. Bigio E. H. Bouras C. Braak H. Cairns N. J.. (2012). Correlation of Alzheimer disease neuropathologic changes with cognitive status: a review of the literature. J. Neuropathol. Exp. Neurol. 71, 362–381. 10.1097/NEN.0b013e31825018f7 22487856 PMC3560290

[B223] Nemeroff C. B. (2016). Paradise lost: the neurobiological and clinical consequences of child abuse and neglect. Neuron 89, 892–909. 10.1016/j.neuron.2016.01.019 26938439

[B224] Nithianantharajah J. Hannan A. J. (2009). The neurobiology of brain and cognitive reserve: mental and physical activity as modulators of brain disorders. Progr. Neurobiol. 89, 369–382. 10.1016/j.pneurobio.2009.10.001 19819293

[B225] Nithianantharajah J. Hannan A. J. (2011). Mechanisms mediating brain and cognitive reserve: experience-dependent neuroprotection and functional compensation in animal models of neurodegenerative diseases. Progr. Neuro-Psychopharmacol. Biol. Psychiatry 35, 331–339. 10.1016/j.pnpbp.2010.10.026 21112312

[B226] Novelo M. von Gunten A. Jardim G. B. G. Spanemberg L. Argimon I. I. L Nogueira E. L. (2018). Effects of childhood multiple maltreatment experiences on depression of socioeconomic disadvantaged elderly in Brazil. Child Abuse Negl. 79, 350–357. 10.1016/j.chiabu.2018.02.013 29522996

[B227] Nummenmaa L. Manninen S. Tuominen L. Hirvonen J. Kalliokoski K. K. Nuutila P.. (2015). Adult attachment style is associated with cerebral μ-opioid receptor availability in humans. Hum Brain Mapp. 36, 3621–3628. 10.1002/hbm.22866 26046928 PMC6869236

[B228] O'Connor E. McCartney K. (2007). Attachment and cognitive skills: an investigation of mediating mechanisms. J. Appl. Dev. Psychol. 28, 458–476. 10.1016/j.appdev.2007.06.007

[B229] Ong A. D. Bergeman C. S. Boker S. M. (2009). Resilience comes of age: defining features in later adulthood. J. Personality 77, 1777–1804. 10.1111/j.1467-6494.2009.00600.x 19807864 PMC2807734

[B230] Orth-Gomér K. Rosengren A. Wilhelmsen L. (1993). Lack of social support and incidence of coronary heart disease in middle-aged Swedish men. Psychosom. Med. 55, 37–43. 10.1097/00006842-199301000-00007 8446739

[B231] Panfile T. M. Laible D. J. (2012). Attachment security and child's empathy: the mediating role of emotion regulation. Merrill-Palmer Q. 58, 1–21. 10.1353/mpq.2012.0003

[B232] Panksepp J. (1998). Affective Neuroscience: The Foundations of Human and Animal Emotions. New York, NY: Oxford University Press.

[B233] Panksepp J. Herman B. Conner R. Bishop P. Scott J. P. (1978). The biology of social attachments: opiates alleviate separation distress. Biol. Psychiatry 13, 607–618. 83167

[B234] Panksepp J. Herman B. H. Vilberg T. Bishop P. DeEskinazi F. G. (1980). Endogenous opioids and social behavior. Neurosci. Biobehav. Rev. 4, 473–487. 10.1016/0149-7634(80)90036-6 6258111

[B235] Panksepp J. Nelson E. Bekkedal M. (1997). Brain systems for the mediation of social separation-distress and social-reward evolutionary antecedents and neuropeptide intermediaries. Ann. N. Y. Acad. Sci. 807, 78–100. 10.1111/j.1749-6632.1997.tb51914.x 9071345

[B236] Pasqualetti G. Brooks D. J. Edison P. (2015). The role of neuroinflammation in dementias. Curr. Neurol. Neurosci. Rep. 15, 1–11. 10.1007/s11910-015-0531-7 25716012

[B237] Pearce E. Wlodarski R. Machin A. Dunbar R. I. M. (2017). Variation in the β -endorphin, oxytocin, and dopamine receptor genes is associated with different dimensions of human sociality. Proc. Natl. Acad. Sci. U.S.A. 114, 5300–5305. 10.1073/pnas.1700712114 28461468 PMC5441808

[B238] Pechtel P. Lyons-Ruth K. Anderson C. M. Teicher M. H. (2014). Sensitive periods of amygdala development: the role of maltreatment in preadolescence. Neuroimage 97, 236–244. 10.1016/j.neuroimage.2014.04.025 24736182 PMC4258391

[B239] Pechtel P. Pizzagalli D. A. (2011). Effects of early life stress on cognitive and affective function: an integrated review of human literature. Psychopharmacology 214, 55–70. 10.1007/s00213-010-2009-2 20865251 PMC3050094

[B240] Perl D. P. (2010). Neuropathology of Alzheimer's diseases. Mount Sinai J. Med. 77, 32–42. 10.1002/msj.20157 PMC2918894 20101720

[B241] Perren S. Schmid R. Herrmann S. Wettstein A. (2007). The impact of attachment on dementia-related problem behavior and spousal caregivers' well-being. Attachment Human Dev. 2, 163–178. 10.1080/14616730701349630 17508315

[B242] Pierrehumbert B. Torrisi R. Ansermet F. Borghini A. Halfon O. (2012). Adult attachment representations predict cortisol and oxytocin responses to stress. Attachment Human Dev. 14, 453–476. 10.1080/14616734.2012.706394 22856618

[B243] Pietrzak R. H. Laws S. M. Lim Y. Y. Bender S. J. Porter T. Doecke J. (2017). Plasma cortisol, brain amyloid-β, and cognitive decline in preclinical alzheimer' s disease: a 6-year prospective cohort study. Biol. Psychiatry Cogn. Neurosci. Neuroimag. 2, 45–52. 10.1016/j.bpsc.2016.08.006 29560886

[B244] Pillai A. G. Arp M. Velzing E. Lesuis S. L. Schmidt M. V. Holsboer F.. (2018). Early life stress determines the effects of glucocorticoids and stress on hippocampal function: electrophysiological and behavioral evidence respectively. Neuropharmacology 133, 307–318. 10.1016/j.neuropharm.2018.02.001 29412144

[B245] Pool L. R. Weuve J. Wilson R. S. Bultmann U. Evans D. A. Mendes de Leon C. F. (2016). Occupational cognitive requirements and late-life cognitive aging. Neurology 86, 1386–1392. 10.1212/WNL.0000000000002569 26984944 PMC4831043

[B246] Popp J. Wolfsgruber S. Heuser I. Peters O. Hüll M. Schröder J.. (2015). Cerebrospinal fluid cortisol and clinical disease progression in MCI and dementia of Alzheimer's type. Neurobiol. Aging 36, 601–607. 10.1016/j.neurobiolaging.2014.10.031 25435336

[B247] Pryce C. R. Rüedi-Bettschen D. Dettling A. C. Weston A. Russig H. Ferger B.. (2005). Long-term effects of early-life environmental manipulations in rodents and primates: potential animal models in depression research. Neurosci. Biobehav. Rev. 29, 649–674. 10.1016/j.neubiorev.2005.03.011 15925698

[B248] Qiu C. Xu W. Fratiglioni L. (2010). Vascular and psychosocial factors in Alzheimer's disease: epidemiological evidence toward intervention. J. Alzheimer Dis. 20, 689–697. 10.3233/JAD-2010-091663 20182015

[B249] Quinlan P. Horvath A. Nordlund A. Wallin A. Svensson J. (2017). Low serum insulin-like growth factor-I (IGF-I) level is associated with increased risk of vascular dementia. Psychoneuroendocrinology 86, 169–175. 10.1016/j.psyneuen.2017.09.018 28963885

[B250] Quirin M. Gillath O. Pruessner J. C. Eggert L. D. (2010). Adult attachment insecurity and hippocampal cell density. Soc. Cogn. Affect. Neurosci. 5, 39–47. 10.1093/scan/nsp042 20007241 PMC2840841

[B251] Ravona-Springer R. Beeri M. S. Goldbourt U. (2012). Younger age at crisis following parental death in male children and adolescents is associated with higher risk for dementia at old age. Alzheimer Dis. Associat. Dis. 26, 68–73. 10.1097/WAD.0b013e3182191f86 21537146 PMC3150597

[B252] Reblin M. Uchino B. N. (2008). Social and emotional support and its implication for health. Curr. Opin. Psychiatry 21, 201–205. 10.1097/YCO.0b013e3282f3ad89 18332671 PMC2729718

[B253] Regev L. Baram T. Z. (2014). Corticotropin releasing factor in neuroplasticity. Front. Neuroendocrinol. 35, 171–179. 10.1016/j.yfrne.2013.10.001 24145148 PMC3965598

[B254] Reincke S. A. J. Hanganu-Opatz I. L. (2017). Early-life stress impairs recognition memory and perturbs the functional maturation of prefrontal-hippocampal-perirhinal networks. Sci. Rep. 7, 1–16. 10.1038/srep42042 28169319 PMC5294456

[B255] Ridderinkhof K. R. Ullsperger M. Crone E. A. Nieuwenhuis S. (2004). The role of the medial frontal cortex in cognitive control. Science 306, 443–448. 10.1126/science.1100301 15486290

[B256] Rifkin-Graboi A. Kong L. Sim L. W. Sanmugam S. Broekman B. F. P. Chen H.. (2015). Maternal sensitivity, infant limbic structure volume and functional connectivity: a preliminary study. Transl. Psychiatry 5:e668. 10.1038/tp.2015.133 26506054 PMC4930120

[B257] Rincón-Cortés M. Sullivan R. M. (2014). Early life trauma and attachment: immediate and enduring effects on neurobehavioral and stress axis development. Front. Endocrinol. 5, 1–15. 10.3389/fendo.2014.00033 24711804 PMC3968754

[B258] Roe C. M. Xiong C. Miller J. P. Morris J. C. (2007). Education and Alzheimer disease without dementia: support for the cognitive reserve hypothesis. Neurology 68, 223–228. 10.1212/01.wnl.0000251303.50459.8a 17224578

[B259] Roque A. Ochoa-Zarzosa A. Torner L. (2016). Maternal separation activates microglial cells and induces an inflammatory response in the hippocampus of male rat pups, independently of hypothalamic and peripheral cytokine levels. Brain Behav. Immun. 55, 39–48. 10.1016/j.bbi.2015.09.017 26431692

[B260] Rosen H. J. Gorno-Tempini M. L. Goldman W. P. Perry R. J. Schuff N. Weiner M.. (2002). Patterns of brain atrophy in frontotemporal dementia and semantic dementia. Neurology 58, 198–208. 10.1212/WNL.58.2.198 11805245

[B261] Rosengren A. Wilhelmsen L. Orth-Gomér K. (2004). Coronary disease in relation to social support and social class in Swedish men. A 15 year follow-up in the study of men born in 1933. Eur. Heart J. 25, 56–63. 10.1016/j.ehj.2003.10.005 14683743

[B262] Rowe A. Carnelley K. B. (2003). Attachment style differences in the processing of attachment-relevant information: primed-style effects on recall, interpersonal expectations, and affect. Personal Relationships 10, 59–75. 10.1111/1475-6811.00036

[B263] Sanders B. J. Anticevic A. (2007). Maternal separation enhances neuronal activation and cardiovascular responses to acute stress in borderline hypertensive rats. Behav. Brain Res. 183, 25–30. 10.1016/j.bbr.2007.05.020 17604851 PMC1994156

[B264] Santini Z. Koyanagi A. Tyrovalas S. Haro J. (2015). The association of relationship quality and social networks with depression, anxiety, and suicide ideation among older married adults: findings from a cross-sectional analysis of the Irish Longitudinal Study on Ageing (TILDA). J. Affect. Dis. 179, 134–141. 10.1016/j.jad.2015.03.015 25863909

[B265] Santos L. Beckman D. Ferreira S. T. (2016). Microglial dysfunction connects depression and Alzheimer' s disease. Brain Behav. Immun. 55, 151–165. 10.1016/j.bbi.2015.11.011 26612494

[B266] Satz P. (1993). Brain reserve capacity on symptom onset after brain injury: a formulation and review of evidence for threshold theory. Neuropsychology 7, 273–295. 10.1037/0894-4105.7.3.273

[B267] Schofield P. W. Logroscino G. Andrews H. F. Albert S. Stern Y. (1997). An association between head circumference and Alzheimer's disease in a population-based study of aging and dementia. Neurology 49, 30–37. 10.1212/WNL.49.1.30 9222166

[B268] Schore A. N. (1996). The experience-dependent maturation of a regulatory system in the orbital prefrontal cortex and the origin of developmental psychopathology. Dev. Psychopathol. 8, 59–87. 10.1017/S0954579400006970

[B269] Schore A. N. (2001). Effects of a secure attachment relationship on right brain development, affect regulation, and infant mental health. Infant Mental Health J. 22, 7–66. 10.1002/1097-0355(200101/04)22:1<7::AID-IMHJ2>3.0.CO;2-N

[B270] Seeman T. Berkman L. Kohout F. Lacroix A. Glynn R. Blazer D. (1993). Intercommunity variations in the association between social ties and mortality in the elderly: a comparative analysis of three communities. Eur. Psychiatry 4, 325–335. 10.1016/1047-2797(93)90058-C 8275207

[B271] Seeman T. E. (2000). Health promoting effects of friends and family on health outcomes in older adults. Am. J. Health Promot. 14, 362–370. 10.4278/0890-1171-14.6.362 11067571

[B272] Seeman T. E. Lusignolo T. M. Albert M. Berkman L. (2001). Social relationships, social support, and patterns of cognitive aging in healthy, high-functioning older adults: MacArthur Studies of Successful Aging. Health Psychol. 20, 243–255. 10.1037/0278-6133.20.4.243 11515736

[B273] Shankar A. Hamer M. McMunn A. Steptoe A. (2013). Social isolation and loneliness: relationships with cognitive function during 4 years of follow-up in the English Longitudinal Study of Ageing. Psychosomat. Med. 75, 161–170. 10.1097/PSY.0b013e31827f09cd 23362501

[B274] Sharp E. S. Gatz M. (2011). The Relationship between education and dementia an updated systematic review. Alzheimer Dis. Assoc. Disord.. 25, 289–304. 10.1097/WAD.0b013e318211c83c 21750453 PMC3193875

[B275] Shorey H. S. Snyder C. R. Yang X. Lewin M. R. (2003). The role of hope as a mediator in recollected parenting, adult attachment, and mental health. J. Soc. Clin. Psychol. 22, 685–715. 10.1521/jscp.22.6.685.22938

[B276] Siedlecki K. L. Salthouse T. A. Oishi S. Jeswani S. (2014). The relationship between social support and subjective well-being across age. Social Indicators Res. 117, 561–576. 10.1007/s11205-013-0361-4 25045200 PMC4102493

[B277] Simpson J. A. (1990). The influence of attachment styles on romantic relationships. J. Personality Soc. Psychol. 59, 971–980. 10.1037/0022-3514.59.5.971

[B278] Simpson J. A. Rholes W. S. (2017). Adult attachment, stress, and romantic relationships. Curr. Opin. Psychol. 13, 19–24. 10.1016/j.copsyc.2016.04.006 28813288

[B279] Simpson J. A. Rholes W. S. Phillips D. (1996). Conflict in close relationships: an attachment perspective. J. Personality Soc. Psychol. 71, 899–914. 10.1037/0022-3514.71.5.899 8939040

[B280] Slavich G. M. Irwin M. R. (2014). From stress to inflammation and major depressive disorder: a social signal transduction theory of depression. Psychol. Bull. 140, 774–815. 10.1037/a0035302 24417575 PMC4006295

[B281] Slavich G. M. Way B. M. Eisenberger N. I. Taylor S. E. (2010). Neural sensitivity to social rejection is associated with inflammatory responses to social stress. Proc. Natl. Acad. Sci. U.S.A. 107, 14817–14822. 10.1073/pnas.1009164107 20679216 PMC2930449

[B282] Snowdon D. A. Kemper S. J. Mortimer J. A. Greiner L. H. Wekstein D. R. Markesbery W. R. (1996). Linguistic ability in early life and cognitive function and Alzheimer's disease in late life: findings from the Nun Study. J. Am. Med. Assoc. 275, 528–532. 10.1001/jama.1996.03530310034029 8606473

[B283] Soldan A. Pettigrew C. Cai Q. Wang J. Wang M.-C. Moghekar A. (2017). Cognitive reserve and long-term change in cognition in aging and preclinical Alzheimer's disease. Neurobiol. Aging 60, 164–172. 10.1016/j.neurobiolaging.2017.09.002 28968586 PMC5679465

[B284] Solms M. Turnball O. (2002). The Brain and the Inner World. London: Karnac.

[B285] Sonntag W. E. Deak F. Ashpole N. Toth P. Csiszar A. Freeman W.. (2013). Insulin-like growth factor-1 in CNS and cerebrovascular aging. Front. Aging Neurosci. 5, 1–14. 10.3389/fnagi.2013.00027 23847531 PMC3698444

[B286] Soztutar E. Colak E. Ulupinar E. (2016). Gender- and anxiety level-dependent effects of perinatal stress exposure on medial prefrontal cortex. Exp. Neurol. 275, 274–284. 10.1016/j.expneurol.2015.06.005 26057948

[B287] Spence R. Jacobs C. Bifulco A. (2018). Attachment style, loneliness and depression in older age women. Aging Mental Health 31, 1–3. 10.1080/13607863.2018.1553141 30596454

[B288] Sperling R. A. Aisen P. S. Beckett L. A. Bennett D. A. Craft S. Fagan A. M.. (2011). Toward defining the preclinical stages of Alzheimer's disease: recommendations from the National Institute on Aging-Alzheimer's Association workgroups on diagnostic guidelines for Alzheimer's disease. Alzheimer Dement. 7, 280–292. 10.1016/j.jalz.2011.03.003 21514248 PMC3220946

[B289] Spitz R. A. (1945). Hospitalism: an inquiry into the genesis of psychiatric conditions in early childhood. Psychoanaly. Study Child 1, 53–74. 10.1080/00797308.1945.11823126 21004303

[B290] Spitz R. A. (1947). De la Naissance á La Parole. Paris: PUF. Trad. 1968

[B291] Sroufe L. A. (1983). Infant-caregiver attachment and patterns of adaptation in preschool: the roots of maladaptation and competence, in Minnesota Symposium in Child Psychology, Vol. 16, ed Perlmutter M. (Hillsdale, NJ: Erlbaum), 41–83.

[B292] Stern Y. (2002). What is cognitive reserve? Theory and research application of the reserve concept. J. Int. Neuropsychol. Soc. 8, 448–460. 10.1017/S1355617702813248 11939702

[B293] Stern Y. (2009). Cognitive reserve. Neuropsychologia 47, 2015–2028. 10.1016/j.neuropsychologia.2009.03.004 19467352 PMC2739591

[B294] Stern Y. (2012). Cognitive reserve in ageing and Alzheimer's disease. Lancet Neurol. 11, 1006–1012. 10.1016/S1474-4422(12)70191-6 23079557 PMC3507991

[B295] Stern Y. Gurland B. Tatemichi T. K. Tang M. X. Wilder D. Mayeux R. (1994). Influence of education and occupation on the incidence of Alzheimer's disease. JAMA 271, 1004–1010. 10.1001/jama.1994.03510370056032 8139057

[B296] Stievenart M. Roskam I. Meunier J. C. Van de Moortele G. (2011). The reciprocal relation between children's attachment representations and their cognitive ability. Int. J. Behav. Dev. 35, 58–66. 10.1177/0165025410370790

[B297] Stoykova R. Matharan F. Dartigues J. F. Amieva H. (2011). Impact of social network on cognitive performances and age-related cognitive decline across a 20-year follow-up. Int. Psychogeriatr. 23, 1405–1412. 10.1017/S1041610211001165 21777501

[B298] Strathearn L. Fonagy P. Amico J. Montague P. R. (2009). Adult attachment predicts maternal brain and oxytocin response to infant cues. Neuropsychopharmacology 34, 2655–2666. 10.1038/npp.2009.103 19710635 PMC3041266

[B299] Sundström A. Westerlund O. Kotyrlo E. (2016). Marital status and risk of dementia: a nationwide population-based prospective study from Sweden. BMJ Open 6:e008565. 10.1136/bmjopen-2015-008565 26729377 PMC4716184

[B300] Taillieu T. L. Brownridge D. A. Sareen J. Afifi T. O. (2016). Childhood emotional maltreatment and mental disorders: results from a nationally representative adult sample from the United States. Child Abuse Neglect. 59, 1–12. 10.1016/j.chiabu.2016.07.005 27490515

[B301] Teicher M. H. Samson J. A. (2016). Enduring neurobiological effects of childhood abuse and neglect. J. Child Psychol. Psychiatry Allied Disciplines 57, 241–266. 10.1111/jcpp.12507 26831814 PMC4760853

[B302] Tilvis R. S. Kähönen-Väre M. H. Jolkkonen J. Valvanne J. Pitkala K. H. Strandberg T. E. (2004). Predictors of cognitive decline and mortality of aged people over a 10-year period. J. Gerontol. Ser. A Biol. Sci. Med. Sci. 59, 268–274. 10.1093/gerona/59.3.M268 15031312

[B303] Tottenham N. (2012). Human amygdala development in the absence of species-expected caregiving. Dev. Psychobiol. 36, 490–499. 10.1002/dev.20531 PMC3404246 22714586

[B304] Tottenham N. Hare T. A. Millner A. Gilhooly T. Zevin J. D. Casey B. J. (2011). Elevated amygdala response to faces following early deprivation. Dev. Sci. 14, 190–204. 10.1111/j.1467-7687.2010.00971.x 21399712 PMC3050520

[B305] Tottenham N. Hare T. A. Quinn B. T. Mccarry T. W. Nurse M. Gilhooly T. (2010). Prolonged institutional rearing is associated with atypically larger amygdala volume and difficulties in emotion regulation. Dev. Sci. 13, 46–61. 10.1111/j.1467-7687.2009.00852.x 20121862 PMC2817950

[B306] Trevarthen C. Aitken K. J. Vandekerckhove M. Delafield-Butt J. Nagy E. (2006). Collaborative regulations of vitality in early childhood: stress in intimate relationships and postnatal psychopathology, in Developmental Psychopathology: Developmental Neuroscience, eds Cicchetti D. Cohen D. J. (Hoboken, NJ: John Wiley and Sons Inc), 65–126. 10.1002/9780470939390.ch2

[B307] Troisi A. Frazzetto G. Carola V. Lorenzo G. Di Coviello M. Siracusano A.. (2012). Variation in the mu-opioid receptor gene (OPRM1) moderates the influence of early maternal care on fearful attachment. Soc. Cogn. Affect. Neurosci. 7, 542–547. 10.1093/scan/nsr037 21742765 PMC3375889

[B308] Uchino B. N. (2006). Social support and health: a review of physiological processes potentially underlying links to disease outcomes. J. Behav. Med. 29, 377–387. 10.1007/s10865-006-9056-5 16758315

[B309] Van Harmelen A. L. Van Tol M. J. Dalgleish T. Van der Wee N. J. A. Veltman D. J. Aleman A.. (2014). Hypoactive medial prefrontal cortex functioning in adults reporting childhood emotional maltreatment. Soc. Cogn. Affect. Neurosci. 9, 2026–2033. 10.1093/scan/nsu008 24493840 PMC4249477

[B310] Van Harmelen A. L. Van Tol M. J. Van Der Wee N. J. A. Veltman D. J. Aleman A. Spinhoven P.. (2010). Reduced medial prefrontal cortex volume in adults reporting childhood emotional maltreatment. Biol. Psychiatry 68, 832–838. 10.1016/j.biopsych.2010.06.011 20692648

[B311] Van IJzendoorn M. H. (1995). Adult attachment representations, parental responsiveness, and infant attachment: a meta-analysis on the predictive validity of the adult attachment interview. Psychol. Bull. 117, 387–403. 10.1037/0033-2909.117.3.387 7777645

[B312] Van Loenhoud A. C. Groot C. Vogel J. W. Van Der Flier W. M. Ossenkoppele R. (2018). Is intracranial volume a suitable proxy for brain reserve? Alzheimer Res. Ther. 10, 1–12. 10.1186/s13195-018-0408-5 30205838 PMC6134772

[B313] Vaughn B. Egeland B. Sroufe L. A. Waters E. (1979). Individual differences in infant-mother attachment at twelve and eighteen months: stability and change in families under stress. Child Dev. 50, 971–975. 10.2307/1129321 535447

[B314] von Gunten A. Bouras C. Kövari E. Giannakopoulos P. Hof P. R. (2006). Neural substrates of cognitive and behavioral deficits in atypical Alzheimer's disease. Brain Res. Rev. 51, 176–211. 10.1016/j.brainresrev.2005.11.003 16413610

[B315] von Gunten A. Miklossy J. Suv,à M. L. Hof P. R. Glannakopoulos P. (2005). Environmental reduplicative paramnesia in a case of atypical Alzheimer's disease. Neurocase 11, 216–226. 10.1080/13554790590944825 16006343

[B316] Wang H. X. Karp A. Winbald B. Fratiglioni L. (2002). Late-life engagement in social and leisure activities is associated with a decreased risk of dementia: a longitudinal study from the Kungsholmen Project. Am. J. Epidemiol. 155, 1081–1087. 10.1093/aje/155.12.1081 12048221

[B317] Wang H. X. MacDonald S. W. S. Dekhtyar S. Fratiglioni L. (2017). Association of lifelong exposure to cognitive reserve-enhancing factors with dementia risk: a community-based cohort study. PLoS Med. 14, 1–17. 10.1371/journal.pmed.1002251 28291786 PMC5349652

[B318] Waters E. Hamilton C. E. Weinfield N. S. (2000). The stability of attachment security from infancy to adolescence and early adulthood: general introduction. Child Dev. 71, 678–683. 10.1111/1467-8624.00175 10953933

[B319] Waters H. S. Waters E. (2006). The attachment working models concept: among other things, we build script-like representations of secure base experiences. Attachment Human Dev. 8, 185–198. 10.1080/14616730600856016 16938702

[B320] Watt D. F. (2017). Reflections on the neuroscientific legacy of Jaak Panksepp (1943–2017), Neuropsychoanalysis 19, 183–198. 10.1080/15294145.2017.1376549

[B321] Watt D. F. Panksepp J. (2009). Depression: an evolutionarily conserved mechanism to terminate separation distress? A review of aminergic, peptidergic, and neural network perspectives. Neuropsychoanalysis 11, 7–51. 10.1080/15294145.2009.10773593

[B322] Weaver I. C. G. Cervoni N. Champagne F. A. D'Alessio A. C. Sharma S. Seckl J. R.. (2004). Epigenetic programming by maternal behavior. Nat. Neurosci. 7, 847–854. 10.1038/nn1276 15220929

[B323] Weinfield N. S. Sroufe L. A. Egeland B. Carlson E. (2008). Individual differences in infant-caregiver attachment: conceptual and empirical aspects of security, in Handbook of Attachment: Theory, Research, and Clinical Applications. Vol. 2, eds Cassidy J. Shaver P. (New York, NY: Guilford Press, 78–101.

[B324] Weinfield N. S. Sroufe L. A. Egeland B. Carlson E. A. (1999). The nature of individual differences in infant–caregiver attachment, in Handbook of Attachment: Theory, Research, and Clinical Applications, eds Cassidy J. Shaver P. R. (New York, NY: Guilford Press, 68–88.

[B325] West K. K. Mathews B. L. Kerns K. A. (2013). Mother–child attachment and cognitive performance in middle childhood: an examination of mediating mechanisms. Early Childhood Res. Q. 28, 259–270. 10.1016/j.ecresq.2012.07.005

[B326] Wilson R. S. Begeny C. T. Boyle P. A. Schneider J. A. Bennett D. A. (2011). Vulnerability to stress, anxiety, and development of dementia in old age. Am. J. Geriatr. Psychiatry 19, 327–334. 10.1097/JGP.0b013e31820119da 21427641 PMC3078621

[B327] Wilson R. S. Krueger K. R. Arnold S. E. Schneider J. A. Kelly J. F. Barnes L. L.. (2007). Loneliness and risk of Alzheimer disease. Arch. General Psychiatry 64, 234–240. 10.1001/archpsyc.64.2.234 17283291

[B328] Winblad B. Palmer K. Kivipelto M. Jelic V. Fratiglioni L. Wahlund L. O. (2004). Mild cognitive impairment—beyond controversies towards consensus. A report of the International Working Group on Mild Cognitive Impairment. J. Int. Med. 256, 240–246. 10.1111/j.1365-2796.2004.01380.x 15324367

[B329] Yang J. Pan P. Song W. Huang R. Li J. Chen K.. (2012). Voxelwise meta-analysis of gray matter anomalies in Alzheimer's disease and mild cognitive impairment using anatomic likelihood. J. Neurol. Sci. 316, 21–29. 10.1016/j.jns.2012.02.010 22385679

[B330] Yang X. D. Liao X. M. Uribe-Mariño A. Liu R. Xie X. M. Jia J. (2015). Stress during a Critical Postnatal Period Induces Region-Specific Structural Abnormalities and Dysfunction of the Prefrontal Cortex via CRF 1. Neuropsychopharmacology 40, 1203–1215. 10.1038/npp.2014.304 25403725 PMC4367464

[B331] Zahodne L. B. Gongvatana A. Cohen R. Ott B. R. Tremont G. (2013). Are apathy and depression independently associated with longitudinal trajectories of cortical atrophy in Mild Cognitive Impairment? Am. J. Geriatr. Psychiatry 21, 1098–1106. 10.1016/j.jagp.2013.01.043 23636003 PMC3797189

[B332] Zahodne L. B. Stern Y. Manly J. J. (2015). Differing effects of education on cognitive decline in diverse elders with low versus high educational attainment. Neuropsychology 29, 649–657. 10.1037/neu0000141 25222199 PMC4362867

[B333] Zeidner M. Endler N. S. (1996). Handbook of Coping: Theory, Research, Applications. Oxford: John Wiley and Sons.

[B334] Zelazo P. D. Craik F. I. Booth L. (2004). Executive function across the life span. Acta Psychol. 115, 167–183. 10.1016/j.actpsy.2003.12.005 14962399

[B335] Zentner G. E. Henikoff S. (2013). Regulation of nucleosome dynamics by histone modifications. Nat. Struct. Mol. Biol. 20, 259–266. 10.1038/nsmb.2470 23463310

[B336] Zimmerman B. J. Schunk D. H. (2001). Self-Regulated Learning and Academic Achievement: Theoretical Perspectives. Mahwah, NJ: Lawrence Erlbaum Associates.

